# Distinct transcriptional signatures in purified circulating immune cells drive heterogeneity in disease location in IBD

**DOI:** 10.1136/bmjgast-2022-001003

**Published:** 2023-02-06

**Authors:** Bram Verstockt, Sare Verstockt, Jonathan Cremer, João Sabino, Marc Ferrante, Severine Vermeire, Padhmanand Sudhakar

**Affiliations:** 1 KU Leuven Department of Chronic Diseases, Metabolism and Ageing, Translational Research Center for Gastrointestinal Disorders (TARGID), IBD group, KU Leuven, Leuven, Belgium; 2 Department of Gastroenterology and Hepatology, University Hospitals Leuven, Leuven, Belgium

**Keywords:** IBD, INFLAMMATORY BOWEL DISEASE, IMMUNOREGULATION

## Abstract

**Objective:**

To infer potential mechanisms driving disease subtypes among patients with inflammatory bowel disease (IBD), we profiled the transcriptome of purified circulating monocytes and CD4 T-cells.

**Design:**

RNA extracted from purified monocytes and CD4 T-cells derived from the peripheral blood of 125 endoscopically active patients with IBD was sequenced using Illumina HiSeq 4000NGS. We used complementary supervised and unsupervised analytical methods to infer gene expression signatures associated with demographic/clinical features. Expression differences and specificity were validated by comparison with publicly available single cell datasets, tissue-specific expression and meta-analyses. Drug target information, druggability and adverse reaction records were used to prioritise disease subtype-specific therapeutic targets.

**Results:**

Unsupervised/supervised methods identified significant differences in the expression profiles of CD4 T-cells between patients with ileal Crohn’s disease (CD) and ulcerative colitis (UC). Following a pathway-based classification (Area Under Receiver Operating Characteristic - AUROC=86%) between ileal-CD and UC patients, we identified MAPK and FOXO pathways to be downregulated in UC. Coexpression module/regulatory network analysis using systems-biology approaches revealed mediatory core transcription factors. We independently confirmed that a subset of the disease location-associated signature is characterised by T-cell-specific and location-specific expression. Integration of drug-target information resulted in the discovery of several new (*BCL6*, *GPR183*, *TNFAIP3*) and repurposable drug targets (*TUBB2A*, *PRKCQ*) for ileal CD as well as novel targets (*NAPEPLD*, *SLC35A1*) for UC.

**Conclusions:**

Transcriptomic profiling of circulating CD4 T-cells in patients with IBD demonstrated marked molecular differences between the IBD-spectrum extremities (UC and predominantly ileal CD, sandwiching colonic CD), which could help in prioritising particular drug targets for IBD subtypes.

**WHAT IS ALREADY KNOWN ON THIS TOPIC**

- Inflammatory bowel disease (IBD) patients with different disease location phenotypes exhibit molecular differences at the primary disease site resulting in varying therapeutic responses.

**WHAT THIS STUDY ADDS**

- Using gene-expression profiles from the blood-derived immune cells of a wide-spectrum of patients with IBD differing by disease location, we identified a core signature segregating ileal Crohn’s disease and UC patients, partly supported by validation at the tissue-level. By integrating drug target and druggability data, we also identified disease location-based subtype specific therapeutic targets.

**HOW THIS STUDY MIGHT AFFECT RESEARCH, PRACTICE OR POLICY**

- Tailored therapies targeting specific subtypes of IBD could help clinicians to overcome the existing therapeutic ceiling prevalent in IBD treatments.

## Introduction

Inflammatory bowel disease (IBD) is a complex disease of the gut, characterised by chronic intestinal inflammation and resulting in reduction in quality-of-life indices.^1^ Besides a complex and multifactorial disease pathogenesis,^2^ IBD incorporates a wide spectrum of clinical phenotypes.^3^ The binary split of IBD into Crohn’s disease (CD) and ulcerative colitis (UC) is primarily based on the macroscopic and microscopic involvement of the gastrointestinal (GI) tract, while CD is characterised by the presence of transmural, but discontinuous inflammation along the entire GI tract, UC is marked by the occurrence of continuous inflammation spreading from the anal margin and is confined to the colonic mucosa.^4^ Additional phenotypical characterisation is primarily based on the Montreal classification, which has limitations and is purely clinical.^4^ Based on emerging evidence, IBD is more and more perceived as a continuous spectrum with UC and ileal CD at both edges of the spectrum.^4^

At a molecular and cellular level, various differences among ileal CD, colonic CD and UC have been revealed: aggregated genetic risk scores representing the cumulative burden of mutations in known IBD risk loci did introduce the concept of a disease spectrum along the disease location axis.^5^ Also, ileal CD is characterised by a distinct T-cell profile comprising Th1 and Th17 cellular subtypes, while colonic CD is more marked by the Th1 profile.^6^

**Figure 1:**
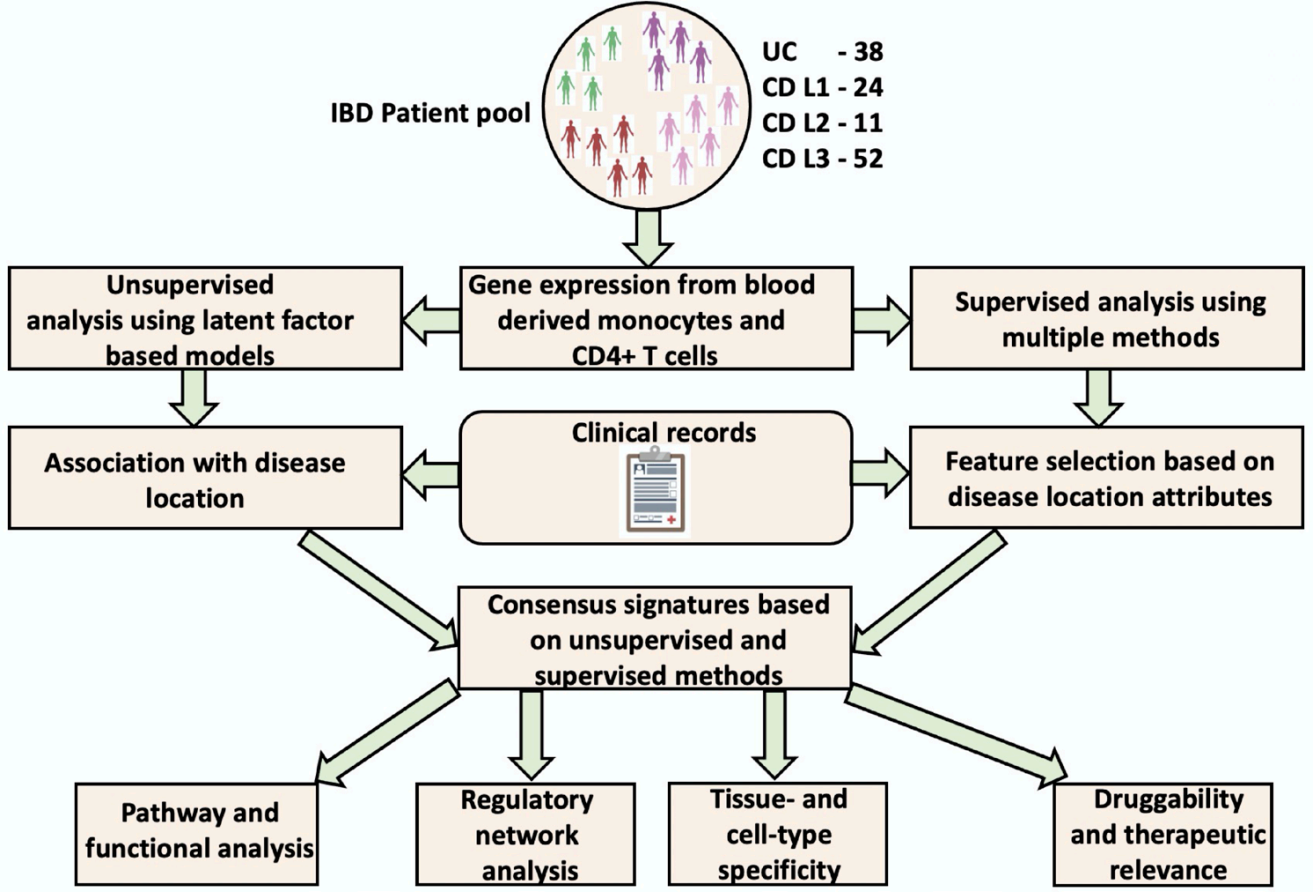
Figurative representation of the workflow used in the study. Unsupervised analysis was carried out using the Multi Omic Factor Analysis (MOFA) tool while the supervised arm of the analysis was executed using DIABLO, netDx, DeSeq2 and BioNERO. Pathway and regulatory network analyses was performed using the ReactomePA package and ChEA3 resource respectively. Tissue-/cell-type specificity and druggability analyses were carried out using publicly available resources such as Human Protein Atlas and OpenTargets/DGIdb/TARDIS respectively. More detailed explanations on the tools used can be found in the Glossary section as well as the methods section of the manuscript. DIABLO, Data Integration Analysis for Biomarker discovery using Latent variable approaches for Omics; DGIdb, Drug-Gene Interaction Database; IBD, inflammatory bowel disease; TARDIS, Target–Adverse Reaction Database Integrated Search.

Purified immune cells from the blood of patients with IBD have previously been used to clinically characterise patients in terms of prognosis^7^ and drug response.^8^ Recent studies have pointed to a significant degree of concordance (between peripheral whole blood and colonic tissue in paediatric patients with IBD.^9^ In addition, blood-based biomarkers have been identified and suggested as surrogate markers of mucosal healing and endoscopic response in Ulcerative colitis^10 11^ as well as predictors of anti-TNFα non-response in IBD.^12^ CD4 T-cells and monocyte-derived macrophages not only play a critical role in mediating disease pathogenesis at the primary disease site(s), but also are known to be altered with respect to their activity in the blood circulatory system.^13–15^ In this study, we use gene expression measurements from circulating monocytes and CD4 T-cells, purified from whole blood, to unravel some molecular differences between patients with IBD with various disease locations. Using a combination of supervised and unsupervised approaches (figure 1), we inferred genes, their modules and regulators which could potentially contribute to the differences observed along the IBD spectrum.

## Methods

### Patient selection

This study was conducted at the University Hospitals Leuven (Leuven, Belgium). Blood was collected from 125 consecutive patients with IBD (24 CD L1, 11 CD L2, 52 CD L3, 38 UC, based on Montreal classification) with endoscopy-proven active disease (presence of ulcerations in case of CD; Mayo endoscopic subscore 2–3 for UC) (online supplemental table 1)

10.1136/bmjgast-2022-001003.supp1 Supplementary data

### RNA sequencing and preprocessing of transcriptomic datasets

Cell separation, fluorescence activated cell sorting, and isolation of RNA were performed as reported earlier.^16^ TruSeq Stranded mRNA protocol (Illumina, San Diego, USA) according to the manufacturer’s instructions was used for the library preparation. Illumina HiSeq 4000NGS was then used for the next-generation single-end sequencing. Alignment of the raw reads from the RNA-sequencing to the reference genome was performed using Hisat2 V.2.1.0.^17^ Absolute counts were subsequently generated using HTSeq.^18^ Only genes with at least 10 reads in at least 70% of the samples were considered. The remaining datasets were subsequently normalised using the varianceStabilizingTransformation function of the daMiRseq R package. A within-sample filtering based on correlation (≥0.85) was used to detect and exclude outliers.

### Multiomic factor analysis

Multiomic factor analysis (MOFA)^19^ was used to identify the contributions of the individual transcriptomic datasets to the overall variance. The top 5000 features as ranked by the mean absolute deviation from each of the two datasets were used as the input for building the MOFA model. Clinical confounder variables such as age, gender, exposure to previous therapies, steroid, immunomodulator and/or biological use were regressed out. DropFactorThreshold was set at 2% in order reflect the selection of latent factors capturing at least 2% variance. Model finetuning was performed by evaluating the performance of 100 random initialisations. The model with the highest evidence lower bound was selected as the best one for further implementation. Latent factors representing at least 5% variance in any one of the datasets were considered as significant and were used for further analyses. The top 1000 features corresponding to the latent factors contributing at least 5% variance were retrieved. Two-tailed Student’s t-test was used to infer relationships among continuous variables.

### Weighted gene coexpression network analysis

A variance filter was applied to select the top 5000 genes from the count filtered normalised dataset. The gene coexpression network was inferred using BioNERO.^20^ Parameters such as the soft threshold was optimised by testing the fitness of the inferred coexpression network to the scale-free topology. Network type was set to unsigned. Module stability was assessed by performing resampling runs (n=30) and assessing the conservation of the modules after every sampling run. The relevance of the modules to the phenotypes (as determined by the association of the module eigengenes to the traits) was assessed to be significant at FDR (False Discovery Rate) ≤0.1. Gene–gene relationships from the phenotypically relevant modules were further filtered by setting the gene–gene Pearson correlation coefficient threshold at 0.6. Hubs were identified by using a combination of two metrics namely module membership and connectivity. Hub genes were defined as the top 10% of the genes with the highest degree which have a module membership of greater than 0.8.

### Differential expression analysis

Pairwise differential expression analysis was carried out using DeSeq2^21^ on the normalised datasets for the different combinations based on disease location. Genes with an absolute log2 fold change of ≥1 and an adjusted p ≤0.1 were considered to be differentially expressed.

### Data Integration Analysis for Biomarker discovery using Latent variable approaches for Omics

For the Data Integration Analysis for Biomarker discovery using Latent variable approaches for Omics (DIABLO)^22^ analysis, the hyperparameter of the design matrix was set at 0.1. The optimal number of components was determined by performing a M-fold validation with 5 folds and 100 repeats. The number of components to be tested for the optimisation run was set at 10. The optimal number of components was selected based on the weighted votes of the various distance measures and error rates. Similarly, the optimal number of features per-omic dataset for each of the components (from the previous step) was determined by running a M-fold validation with 5 folds and 100 repeats. The final model was executed based on the optimised number of components and the number of features per block per component.

### netDx

netDx^23^ was used to build a pathway-based classifier with the input being the normalised gene expression datasets labelled with the corresponding phenotypes. The classifier was built using a set value of 100 for the number of iterations. Maximum feature score was set at 10 with a minimum feature selection score threshold set at 9. Training data were assigned as 70% of the corresponding starting dataset. Only features which passed the minimum feature selection score threshold for at least 90% of the iterations were considered as significant. The aggregated signalling pathways (http://download.baderlab.org/EM_Genesets/March_01_2021/Human/, version March 2021) was used as the feature source to project the gene expression data.

### Functional enrichment analysis

The ReactomePA^24^ and clusterProfiler^25^ R packages were used to check for the presence of over-represented Reactome signalling pathways. Correction for multiple testing was performed with the Benjamini-Hochberg method. Reactome pathways with an FDR≤0.1 were deemed significant. Functional terms with ≤10 entities were discarded from the analysis.

### Regulatory network reconstruction

Transcriptional regulatory networks driving the expression of the phenotypically relevant coexpression modules were reconstructed by integrating the edges within the respective modules with ChIP-seq and independent coexpression datasets from high-throughput measurements. ChIP-seq and independent coexpression relationships corresponding to the two phenotypically relevant gene modules identified using WGCNA^26^ were retrieved from ChEA3,^27^ which is an orthogonal evidence-based resource to identify regulatory and gene–gene relationships. From within the ChEA3 resource, ChIP-seq datasets were retrieved from the parent sources (ENCODE,^28^ ReMap^29^ and literature) whereas additional coexpression data were downloaded from GTEx^30^ and ARCHS4.^31^ An edge (gene–gene relationship) from the phenotypically relevant coexpression module was annotated as a regulatory relationship if one of the genes of the edge is a transcription factor capable of binding to the cis-regulatory elements of the other gene comprising the edge. In addition, if the genes corresponding to the above inferred regulatory relationship were identified as being coexpressed based on independent inferences as recorded in the GTEx and ARCHS4 databases, then the regulatory relationship was annotated with this extra information which could enhance the probability of it being a true regulatory interaction.

### Tissue-type and cell-type origins

Tissue-type and cell-type specific information was retrieved from the Human Protein Atlas.^32^ Single cell transcriptomic datasets were confined to those genes, which were annotated to be ‘group-enriched’, ‘cell-type enriched’ or ‘cell-type enhanced’. As for blood cell specificity in terms of gene expression, genes ‘not detected in immune cells’ or having ‘low immune cell specificity’ were discarded. Similarly, for RNA blood lineage specificity in terms of gene expression, genes ‘not detected’ in blood cells or having ‘low lineage specificity’ were discarded. Single cell transcriptomic and RNA blood lineage specificity datasets corresponding to the category of ‘T-cells’ were retrieved while those of the available and relevant T-cell subtypes (‘memory CD4 T-cells’, ‘naive CD4 T-cells’ and ‘T-regs’) were used in case of blood cell specific gene expression. Gene expression differences between UC patients and non-IBD controls were retrieved from a previously published meta-analysis using eight different datasets.^33^

### Disease and therapeutic relevance

Drug targets used in IBD, and other IMIDs such as rheumatoid arthritis (RA), primary sclerosing cholangitis (PSC), ankylosing spondylitis (AS) and psoriasis (PS) were derived from OpenTargets,^34^ whereas IBD relevant genes corresponding to barrier function,^35^ antimicrobial peptides,^36^ cell-adhesion molecules^37^ and IBD susceptibility loci^38^ were retrieved from the corresponding literature sources. IBD eQTLs were retrieved from Di Narzo *et al*.^39^ Druggability information was retrieved from Drug–Gene Interaction Database (DGIdb),^40^ while adverse drug reaction records corresponding to drug targets were obtained from the Target-Adverse Reaction Database Integrated Search platform which stores integrated records collected from various individual databases such as Drug Target Commons,^41^ STITCH,^42^ FAERS FDA Adverse Event Reporting System,^43^ MedEffect,^44^ Side Effect Resource^45^ and Offsides.^46^ Expression signature-based screening against drug induced expression libraries were performed against the LINCS database.^47^

## Results

### Unsupervised analysis identifies prominent drivers and pathways of IBD pathogenesis

**Figure 2:**
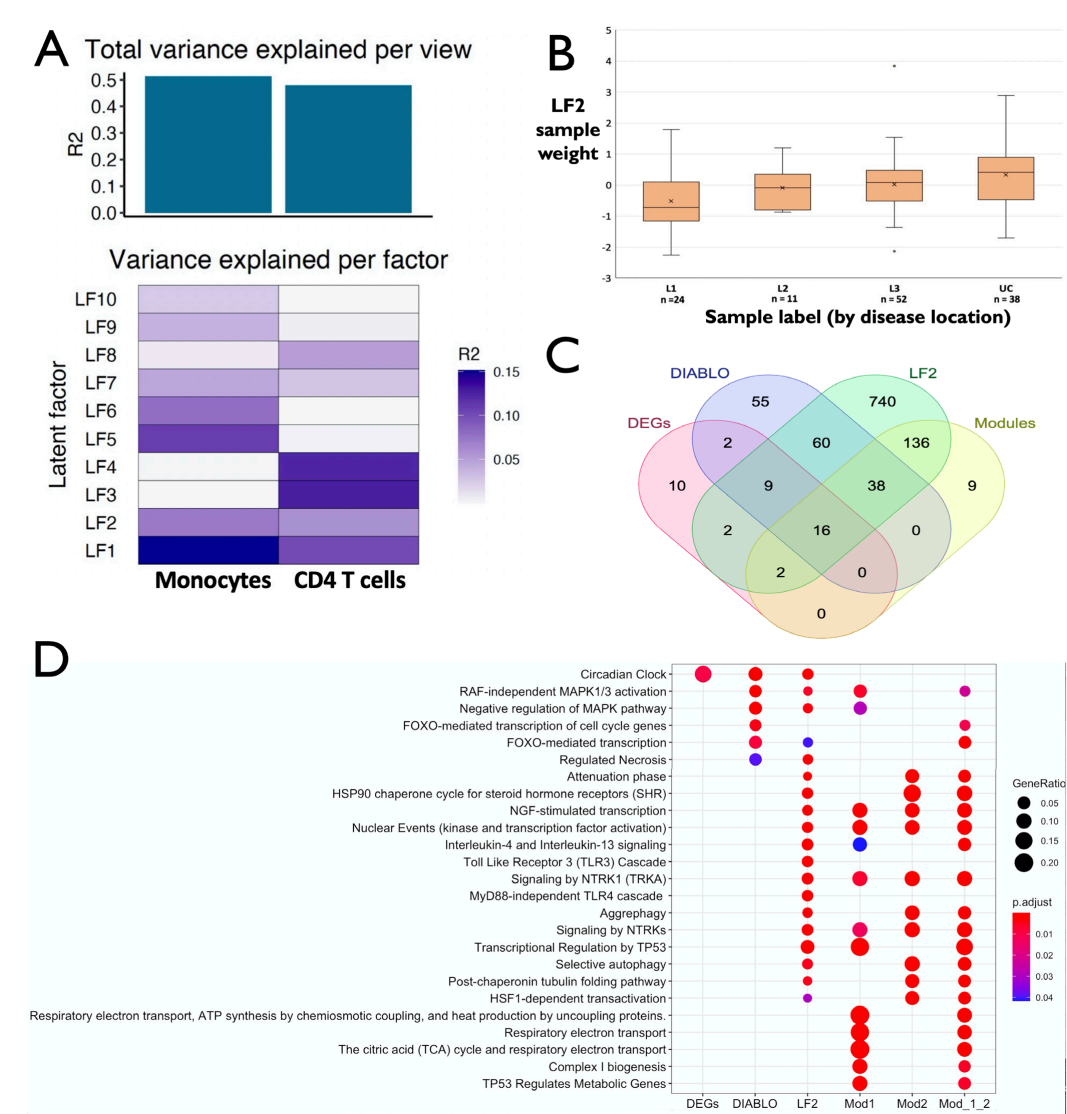
(A) Fraction of total variance explained by the individual latent factors per dataset and proportion of total variance captured cumulatively by the CD4 and monocyte gene-expression datasets. (B) Weights assigned by LF2 to disease location/diagnosis segregated samples. (C) Overlap among the CD4 T cell derived gene expression signatures distinguishing UC and ileal CD patients, inferred from multiple supervised and unsupervised approaches. LF2, Latent factor 2 (associated with disease location) as inferred by MOFA; Modules: union of the two gene coexpression modules associated with disease location. (D) Comparison of over-represented pathways within signature gene sets segregating UC and ileal CD gene expression in CD4 T-cells. Mod_1_2: Union sum of the modules 1 and 2 associated with the disease location-based phenotypic axis segregating UC and ileal CD patients. CD, Crohn’s disease; DEGs, differentially expressed genes; DIABLO, Data Integration Analysis for Biomarker discovery using Latent variable approaches for Omics studies; UC, ulcerative colitis.

Before harnessing supervised approaches, we used MOFA, an unsupervised methodology, to infer genes with highly variant expression patterns. Unsupervised analysis aids in the identification of these underlying patterns in a large dataset, which subsequently can be investigated for their association with relevant clinical phenotypes. Results from the unsupervised integration of the two gene-expression datasets (figure 2A, online supplemental figure 1) pointed to two (LF1, LF2) latent factors, (analogous to components of a principal component analysis, PCA) which capture at least 5% variance from both the datasets. For each of the two cell types, comparative analysis of the features as ranked by the weights of the LFs indicated the presence of unique cell-type specific signatures in each LF. Based on the weights (used to rank the features) which are representative of the variance in expression levels, the top-features identified in each of the two cell-types (online supplemental tables 2–6, online supplemental figure 1) capture key drivers in the monocytes and CD4 T-cells of patients with IBD. Among those are *IL1B*, *CD69*, *OSM*, *GOS2*, *IL8*, *CD83* in monocytes, which all have established roles in IBD pathophysiology and/or intestinal inflammation. Similarly, top hits in the CD4 T-cell dataset including *NR4A2*, *SLC2A3*, *SMAD7*, *TFNAIP3*, *RGS1*) have previously been reported in the context of IBD (table 1, online supplemental tables 2–6, online supplemental figure 1).

10.1136/bmjgast-2022-001003.supp9 Supplementary data

10.1136/bmjgast-2022-001003.supp2 Supplementary data

10.1136/bmjgast-2022-001003.supp3 Supplementary data

10.1136/bmjgast-2022-001003.supp4 Supplementary data

10.1136/bmjgast-2022-001003.supp5 Supplementary data

10.1136/bmjgast-2022-001003.supp6 Supplementary data

**Table 1:** Top ranking features of the monocytes CD4 T cell gene expression dataset as weighted by the latent factor LF2 which is associated with disease location

Gene symbol	Latent factor (dataset)	Encoded protein	IBD relevant function	Reference
NR4A2	LF2 (CD4 T cells)	Nuclear receptor subfamily 4 group A member 2	Imparts protection against IBD by silencing TRAF6/TLR-IL-1R signalling	72
CSRNP1	LF2 (CD4 T cells)	Cysteine/serine-rich nuclear protein 1	–	–
SLK1	LF2 (CD4 T cells)	Serine/threonine-protein kinase SIK1	Identified as a possible drug target for colitis based on small-molecule screening	73
FOSB	LF2 (CD4 T cells)	Protein fosB	–	–
NR4A1	LF2 (CD4 T cells)	Nuclear receptor subfamily 4 group A member 1	Susceptibility loci in a Japanese family with CD; Modulation of inflammation-associated intestinal fibrosis	74 75
NR4A3	LF2 (monocytes)	Nuclear receptor subfamily 4 group A member 3	–	–
IL1B	LF2 (monocytes)	Interleukin-1 beta	Increases intestinal tight junction permeability	76 77
CD69	LF2 (monocytes)	Early activation antigen CD69	Modulates mucosal inflammation in patients with IBD	78
PDE4B	LF2 (monocytes)	cAMP-specific 3',5'-cyclic phosphodiesterase 4B	PDE4 Inhibition confers protection against UC	79
OSM	LF2 (monocytes)	Oncostatin-M	Drives intestinal inflammation and predicts response to tumour necrosis factor-neutralising therapy; Mediates STAT3-dependent intestinal epithelial restitution; Predicts Crohn’s disease response to Infliximab; Biomarker of poor biochemical response to Infliximab	80–84

Only the top five features are shown along with their roles in IBD pathogenesis and/or intestinal inflammation.

CD, Crohn’s disease; IBD, inflammatory bowel disease; UC, ulcerative colitis.

Subsequently, we investigated the association between LFs and enrichment of clinical variables. Only LF2 displayed a significant association (R^2^=0.28, FDR=0.012) with disease location, but did not correlate with any other clinically relevant variables (disease duration, CRP) tested, indicating its specificity with respect to disease location. Although sample clustering based on the weights assigned by LF2 did not result in a perfect segregation based on disease location status (along the phenotypic axis of L1, L2, L3, UC), we observed a significant difference (p=0.003) in the scores assigned by LF2 to L1 and UC samples (figure 2B).

### Orthogonal supervised approaches recapitulate consensus signatures captured by the strongest latent factors and segregate UC and CD ileal subtypes

To complement our observations from the unsupervised approach, we performed supervised analysis using three independent tools (DeSeq2, DIABLO and netDx), which aids the inference of consensus signatures inferred by more than any single tool or method and hence enriching the interpretations. Irrespective of the tool used and in line with the unsupervised analysis, we could identify signatures in CD4 T-cells (figure 2C), which segregated UC from CD L1 individuals (and not comparisons between other combination of samples differing by disease location) through circulating CD4 T cell transcriptomics. To further characterise the signatures from each tool, we compared the functional content in terms of the over-represented signalling pathways (figure 2D). In addition, we also performed a coexpression network analysis (online supplemental figure 2) to confirm the inferences made using the three above-mentioned supervised tools.

10.1136/bmjgast-2022-001003.supp10 Supplementary data

**Figure 3:**
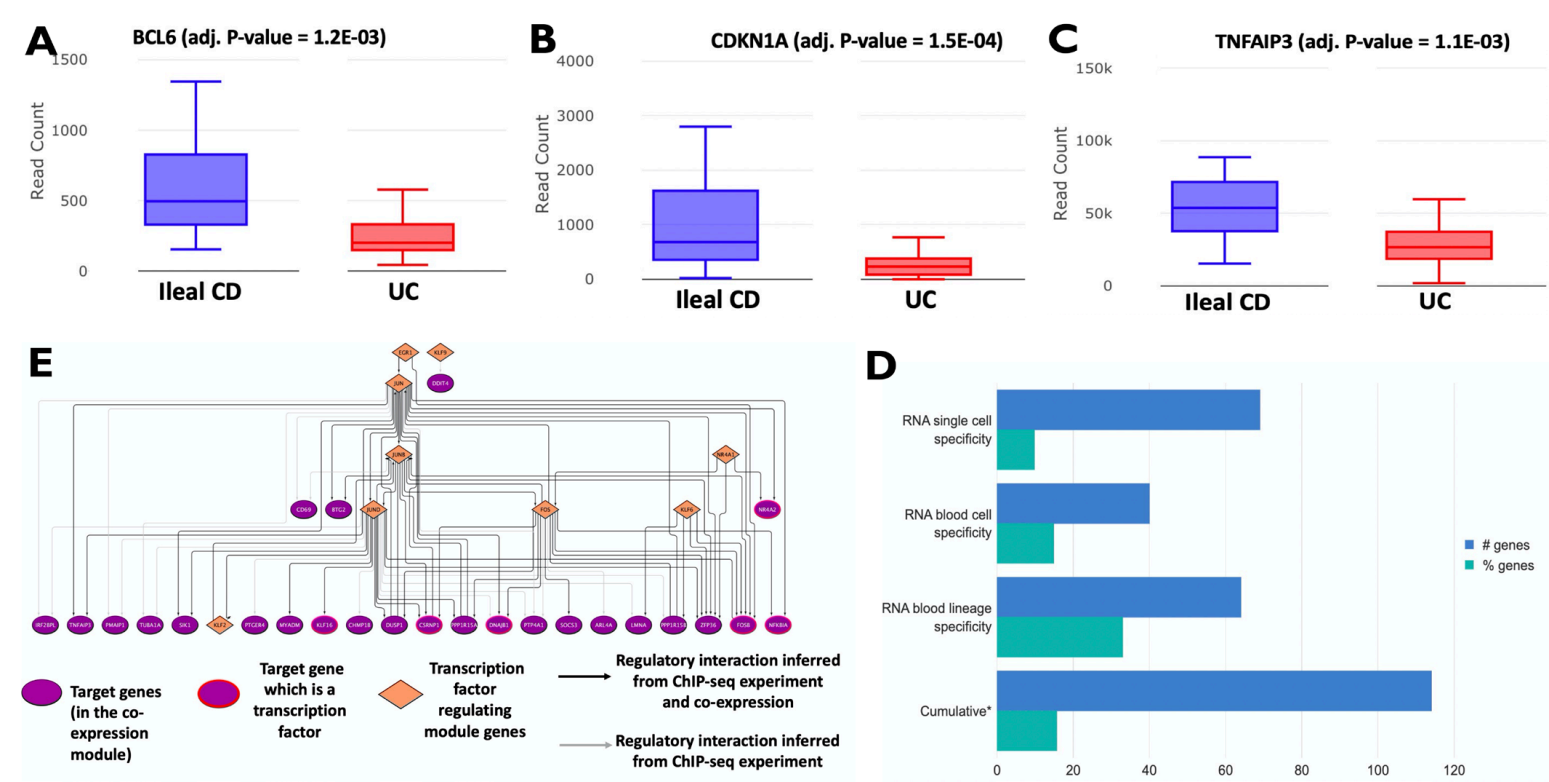
(A, B) Difference in the transcriptional expression between ileal CD and UC patients of FOXO-pathway members *BCL6* and *CDKN1A*. (C) Difference in the transcriptional expression between ileal CD and UC patients of TNFAIP3. (D) Summary of cell-type specific expression profiles for the disease location (ileal CD vs UC) associated signature. Using publicly available gene expression datasets at the level of single cells and beyond from the Human Protein Atlas (HPA), we inferred the cell-type specificity of the disease location-associated genes. Essentially, the figure represents the overlap between the disease location (ileal CD vs UC) associated genes in CD4 T cells and (D) Genes expressed in various T-cell populations as inferred by RNA sequencing at the resolution of single cells (RNA single cell specificity) irrespective of sample source, genes expressed in various blood-derived T-cell populations (RNA blood cell specificity), genes expressed in blood-derived T-cell populations at the level of cell-lineages (RNA blood lineage specificity). (*) The cumulative number is the non-redundant sum of all of the above categories. The numbers are represented both as a fraction and absolute gene count of all the disease location-associated genes. Please refer to the Methods section for more details on the filtering procedure used. (E) Transcriptional regulatory network modulating the expression of the coexpression module 1 associated with disease location. CD, Crohn’s disease; UC, ulcerative colitis.

While DeSeq2 produced the most conservative signature in the form of differentially expressed genes (DEGs) between L1 and UC (online supplemental figure 3), the CD4 T cell signature set from DIABLO (online supplemental figure 4), the strongest latent factor from MOFA (LF2) as well as the two phenotypically relevant coexpression modules (online supplemental figures 2, 5) were enriched with multiple pathways. Some including the RAF-independent MAPK-activation pathway and FOXO-mediated transcriptional pathway (figure 3A–C, online supplemental figure 6) were over-represented in multiple signature sets derived from different approaches. Pathway-level classification using the netDx classifier resulted in an AUROC of 86% (online supplemental figures 7, 8) between ileal CD and UC patients. The features which drive the segregation between the two patient groups include pathways such as FOXO-mediated signalling, p38(MAPK)-mediated signalling among others.

10.1136/bmjgast-2022-001003.supp11 Supplementary data

10.1136/bmjgast-2022-001003.supp12 Supplementary data

10.1136/bmjgast-2022-001003.supp13 Supplementary data

10.1136/bmjgast-2022-001003.supp14 Supplementary data

10.1136/bmjgast-2022-001003.supp15 Supplementary data

10.1136/bmjgast-2022-001003.supp16 Supplementary data

To further unravel the blood-derived signals associated with disease location, we investigated their cell and tissue specificity. About 16% of the genes within the disease location-based CD4 T-cell signature (with available single cell transcriptomic data) are expressed in T-cells (figure 3D, online supplemental figure 9). Meanwhile, the equivalent measure for blood-derived T-cells was determined to be about 33%, pointing to a certain level of cell-type specificity of our disease location-associated CD4 T-cell signature segregating ileal CD and UC patients. However, only a small fraction (4.2%) of the genes in CD4 T-cell signature could be traced to intestinal origins based on their expression.

10.1136/bmjgast-2022-001003.supp17 Supplementary data

### Regulatory networks driving CD4 T cell gene expression signatures reveal a core set of master transcription factors

To infer the control mechanisms which drive the expression of phenotypically relevant genes, we used experimentally verified transcriptional regulatory and coexpression networks. The correlational nature of coexpression networks and the mechanistic knowledge imparted by transcriptional interactions enables the integration of contextual signals with the underlying cis-regulatory wiring. As a first step, we detected highly inter-connected gene sets representing groups of genes with similar expression patterns (online supplemental figures 2, 5). Of the 14 modules detected, only 2 were significantly associated (FDR≤0.1) with disease location. Functional enrichment analysis of the modules not only revealed genes corresponding to prominent pathways such as the MAPK pathway and IL-4/IL-13 signalling, for example, in module 1, but also of various modulators and accessory molecules (such as CDKN1A (figure 3E), which influences the activity of the MAPK pathway). More than two-thirds (9/13) of the enriched pathways in module 1 were unique to module 1 compared with module 2. Enriched pathways common to both the modules include those related to NTRK signalling and NGF-stimulated transcription (figure 2D). Top hubs of the coexpression network include *PPP1R15A*, TUBA1A, *CSRNP1*, *FOSB* and *ZFP36* in module 1 and *TUBB4B, SERTAD1, FAM53C, PLK2* and *HEXIM1* in module 2.

Since gene coexpression networks representing gene–gene relationships were shown to capture context specificity, we used the phenotypically relevant coexpression modules to further infer the underlying mechanistic interactions such as regulatory relationships by annotating the module edges with ChIP-seq data derived from high-throughput experiments and independent coexpression measurements. Seventy-five per cent (55/73) of the edges within the coexpression module 1 associated with disease location were characterised by regulatory binding, as well as independent coexpression relationships (figure 3E). Nodes within the coexpression module 1 encoded for biologically relevant proteins such as *SOCS3*, *NFKB1A*, *TNFAIP3* (figure 3A–C) among others. Furthermore, this coexpression module was regulated by multiple core transcription factors of the JUN family, *FOS*, *NR4A1*, *EGR1* and three TFs (*KLF2*, *KLF6*, *KLF9*) from the Krueppel-like factor family of transcription factors. The abovementioned TFs also regulated various other transcription factors such as *NR4A2*, *NFKB1*, *KLF16*, *DNAJB1* and *CSRNP1*, all found within the same coexpression module. Despite its low connectivity, *EGR1* acts as a master regulator by modulating the expression of upstream transcription factors such as *JUN*. Module 2 on the other hand was characterised by a sparse regulatory network with a low number of inferred interactions (data not shown).

### Therapeutic and disease relevance of immune-cell-derived molecular targets associated with disease location

Functional relevance of the inferred signatures is essential for biological interpretation and shortlisting targets for therapeutic interventions. We; therefore, compared the genes (and/or their expression) making up our disease location-associated signature with (A) previously identified IBD-relevant genes, (B) targets of drugs used in IBD and other IMIDs and (C) by exploring the druggability and adverse reactions of hitherto unexplored targets. For the above analysis, we used the set of genes which were identified by at least two methods (from among MOFA, DEGs, DIABLO and coexpression analysis).

**Figure 4:**
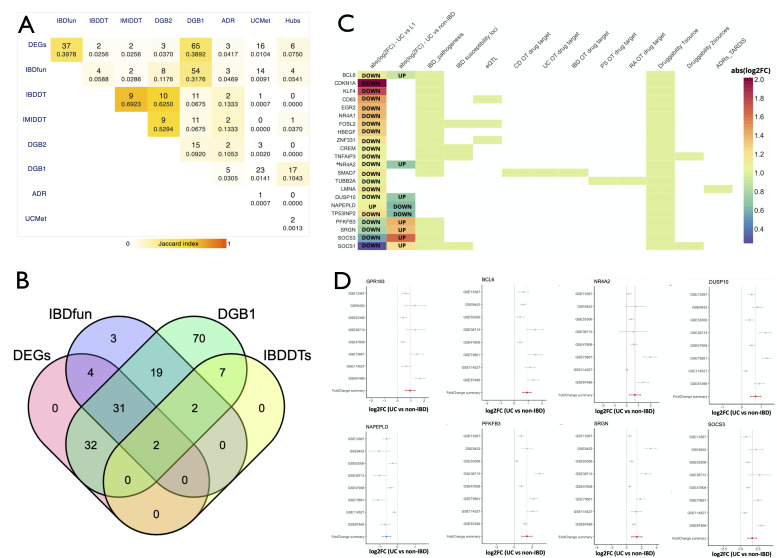
(A) Correlation matrix representing the overlap profile (of the disease location associated signature) between the gene categories involved in IBD pathogenesis (IBDfun), therapeutically targeted in IBD (IBDDTs), in IMIDs (IMIDDTs), those identified as druggable targets (DGB1 and DGB2 for targets assigned as druggable by DGIdb based on one and two sources, respectively) and those with adverse drug reactions (ADR). DEGs: differentially expressed genes (UC vs L1) at log2 fold change ≥0.5 and at an adjusted p≤0.1; UCMeta: genes differentially expressed (as identified by a meta-analysis) in the intestinal mucosa between UC and non-IBD controls. (B) Disease location signature genes distributed with respect to their role in IBD pathogenesis (IBDfun), status in terms of being therapeutically targeted in IBD (IBDDTs), druggability (DGB1) and differential expression (log2 fold change ≥0.5 and adjusted p≤0.1) between UC vs L1 patients. (C) IBD pathogenesis and therapeutic relevance of the CD4 T cell signature (differentiating ileal CD and UC patients) associated with disease location. abs (log2FC) – UC vs L1: absolute fold change difference of gene expression between UC and ileal CD patients; abs (log2FC) – UC vs non-IBD: absolute fold change difference of gene expression between UC patients and non-IBD controls as inferred from a meta-analysis^33^; AMP genes : genes encoding anti-microbial peptides; IBD susceptibility loci : susceptibility genes identified by^38^; eQTL: expression based Quantitative Trait Loci (QTL) retrieved from Di Narzo *et al*.^39^ CD OT drug target: drug targets used in Crohn’s disease as identified from the OpenTargets database^34^; UC OT drug target : drug targets used in Ulcerative colitis as identified from the OpenTargets database^34^; IBD OT drug target: drug targets used in IBD as identified from the OpenTargets database^34^; PS OT drug target : drug targets used in psoriasis disease as identified from the OpenTargets database^34^; RA OT drug target : drug targets used inRA disease as identified from the OpenTargets database^34^; Druggability—2 sources : targets identified as ‘druggable’ by at least two sources in the Drug Gene Interaction database DGIdb^40^; Druggability—1 sources : targets identified as ‘druggable’ by at least one source in the Drug Gene Interaction database DGIdb^40^; ADR record: information as recorded in Target–Adverse Reaction Database Integrated Search (TARDIS). Genes with evidence from each of above-mentioned classes were filtered based on the absolute log2 fold change ≥1 in one of the two expression datasets (UC vs L1 OR UC vs non-IBD) and sorted; *annotation of the genes in terms of their identification as a hub in the coexpression modules associated with disease location. (D) Expression differences between UC patients and non-IBD controls (based on the results of a meta-analysis) for some of the candidate disease location associated genes with therapeutic potential. ADR, adverse drug reaction; CD, Crohn’s disease; IBD, inflammatory bowel disease; RA, rheumatoid arthritis; UC, ulcerative colitis.

To substantiate the functional importance of the two phenotypically relevant CD4 T cell signature associated with disease location, the signature genes were matched against pre-existing gene sets corresponding to IBD drug targets,^34^ IBD relevant genes (barrier function,^35^ antimicrobial peptides,^36^ cell-adhesion molecules,^37^ IBD susceptibility loci,^38^ literature search) and IBD eQTLs^39^ (figure 4A). About 23% (61/265) of the disease location-associated signature genes were linked to IBD pathogenesis (online supplemental table 6) and 28/61 were also annotated as eQTLs. In total, only 4 (*CXCR4, PRKCQ, PTGER4* and *SMAD7*) of the 57 disease location-associated genes relevant to IBD pathogenesis were known to be therapeutic targets in IBD, signifying the discovery of novel and additional targets with therapeutic potential (figure 4A–B, online supplemental table 6). Fifty genes were further identified as druggable based on annotation (from only one parent resource of druggability from the DGIdb (figure 4A–B, online supplemental table 6). By raising the druggability threshold to two sources, the number of potential druggable targets is reduced to eight (*TNFAIP3*, *PTGER4*, *GPR183*, *CXCR4*, *NR1D2*, *PRKCQ*, *SLC30A1* and *SOCS1*).

5.6% of the disease location signature genes were identified as druggable (online supplemental table 6). These include UC-downregulated genes encoding proteins such as *TNFAIP3* (absolute log2FC UC vs L1=−1.06; FDR=0.001; figure 3C), *PTGER4, GPR183, NR1D2, SIRT1* and *PRKCQ*. Of these, *SIRT1* and *PTGER4* were identified to have potential adverse reactions when targeted (online supplemental table 6). However, genes (*BCL6*, *CDKN1A*, *BTG1*, *GADD45A*, *KLF4*) (figure 3A–C) within the downregulated FOXO-pathway (which was one among the pathways consistently found to be over-represented among feature sets inferred by multiple methods) have not yet been targeted in IBD as well as other IMIDs (PS, RA, SC and SA). Although many of the disease location-associated genes with the strongest expression differences between UC and ileal CD patients were unsurprisingly involved in IBD pathogenesis, they present a potentially rewarding pool of therapeutic targets (figure 4C, online supplemental table 6). Examples include *TUBB2A* which is targeted in PS and RA and others such as *BCL6, NR4A2, DUSP10, NAPEPLD, PFKFB3, SRGN, SOCS3* and *SOCS1* which also exhibit differences between UC and non-IBD controls. Surprisingly, most of the (disease location signature) genes (20/24) with significant expression differences in the meta-analysis between UC patients and non-IBD controls displayed the opposite expression fold changes (downregulated in UC vs L1 and upregulated in UC vs non-IBD) (figure 4D).

As a complementary method, we also used the magnitude of the expression differences between ileal CD and UC patients to predict potential drugs for the specific subtypes of IBD defined by disease location. A reverse-effect screen (online supplemental table 7, online supplemental figure 10) resulted in the identification of possible drugs with a higher score (representative of the ability to modulate the expression signature) of 18.004 than a mimic-effect screen (0.294) (online supplemental figure 11, online supplemental table 8). Prominent modulators in the reverse-effect screen include agents such as withaferin-a, celastrol, narciclasine, ryuvidine, curcumin and mocetinostat among others (online supplemental table 7).

10.1136/bmjgast-2022-001003.supp7 Supplementary data

10.1136/bmjgast-2022-001003.supp18 Supplementary data

10.1136/bmjgast-2022-001003.supp19 Supplementary data

10.1136/bmjgast-2022-001003.supp8 Supplementary data

## Discussion

Disease location in patients with IBD carries a high degree of interpatient variability,^4^ although within a given patient it seems to be imprinted and genetically determined.^5^ Despite the underlying mechanisms which mediate location-specific phenotypic manifestations occur along the intestinal tract (the primary disease site), circulating immune cells and their expression signatures have been used to predict disease and treatment outcomes.^48^ In the current study, we performed transcriptomic profiling of monocytes and circulating CD4 T-cells purified from whole blood retrieved from CD and UC patients. Using a combination of supervised and unsupervised approaches, we investigated its link with disease location. Indeed, blood-derived CD4 T cell transcriptomics shed light on the two extremes of the IBD spectrum: UC and ileal CD with no significant differences observed between other sample groups with respect to disease location.

**Figure 5:**
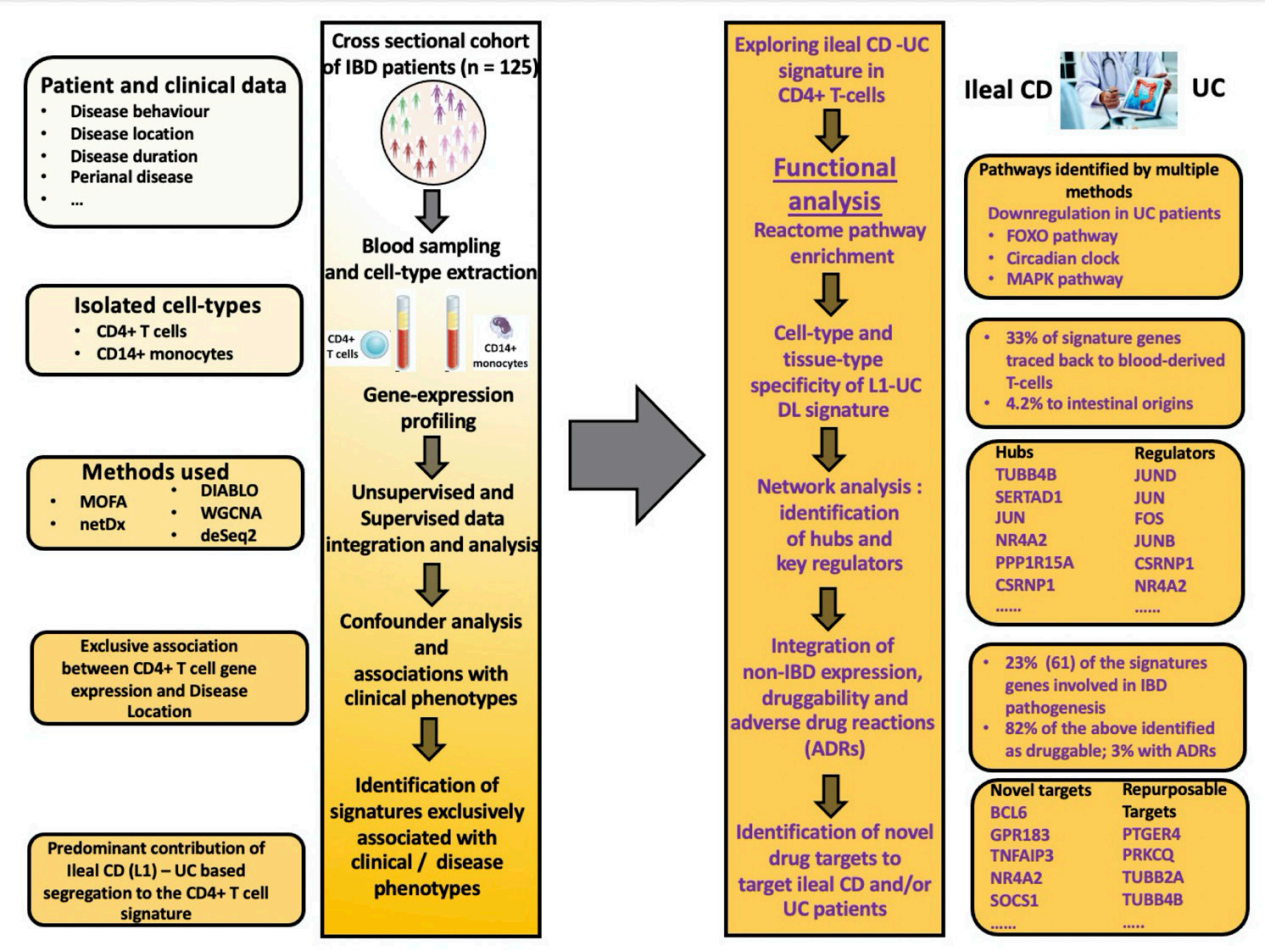
Summary of the workflow and results from the study. CD, Crohn’s disease; IBD, inflammatory bowel disease; UC, ulcerative colitis.

Instead of using a single methodology, we applied multiple methods (supervised and unsupervised) with different underlying theoretical bases to confirm whether results from different methods could converge into a common set of phenotypically relevant signals (figure 5). While differential expression analysis using DeSeq2 identified genes with a substantial variation in the mean expression between the ileal CD and UC patients, it does not consider codependencies such as coexpression of genes within a pathway or coexpression of genes between cell types. Hence, pathways enriched within the set of DEGs are expectedly sparse, and therefore provide little ground for comparison. In contrast, alternative methods (DIABLO) take into account not only the segregation of samples, but also their correlation with features from adjacent datasets (ie, the two gene expression datasets from the different cell types in our case). BioNERO, meanwhile, relies on the similarity of expression profiles among genes within the same dataset to infer modules of coexpressed genes. MOFA, by virtue of being an unsupervised method, identifies higher-order latent factors (equivalent to principal components in a PCA) representative of the expression profiles of the genes in each of the datasets. By associating the latent factors (via the weights assigned to the samples) to the clinical heterogeneity variables, we identified ‘explanatory’ latent factors. At the level of genes, about a quarter (24.55%) of the disease location-associated genes identified cumulatively were inferred by at least two different methods, thus bringing out the complementarity between the methods. As an additional validation step, we also compared the above-described signature to DEG-sets representing three distinct contexts at the tissue level (A) CD L1 inflamed ileum vs control ileum (B) UC inflamed colon vs control colon and (C) CD L1 inflamed ileum versus UC inflamed colon. Eventhough the validation datasets were not derived from the same cohort of patients, 44% of the DEGs (in CD4 T cells) distinguishing CD L1 and UC patients were also modulated in at least one validation dataset (online supplemental figure 12).

10.1136/bmjgast-2022-001003.supp20 Supplementary data

Additionally, at the level of pathways, we observed several ones which are over-represented within the disease location-associated gene sets identified by multiple methods. For example, pathway enrichment analysis within the circulating CD4 T cell transcriptome highlighted MAPK-associated pathways, FOXO-pathways in the differentiation between UC and ileal CD, which simultaneously popped-up through additional unsupervised and supervised analytical approaches and was further confirmed by coexpression-based gene modules. Although no direct comparisons have previously been made between ileal CD and UC patients based on peripheral gene expression, the modulation of the MAPK pathways in UC is supported by earlier experimental studies.^49–51^ In contrast with all the aforementioned supervised methods, netDx uses the underlying pathway structures as meta-features to interpret and integrate the gene expression signals from the same dataset. In agreement with the over-representation of MAPK and FOXO pathways among the other supervised methods, netDx also captured the same pathways among the meta-level features (AUROC=86%) segregating ileal CD and UC patients.

Another discriminative pathway which was identified as a prominent feature by multiple methods is the FOXO transcriptional pathway, which based on our data seems key in ileal CD patients. The FOXO family of transcription factors such as FOXO3 are implicated in IBD pathology^52^ and the regulation of intestinal homoeostatic processes such as inflammation, autophagy, mucus secretion, microbe–host interactions and maintenance of the intestinal barrier integrity.^53–56^ GWAS data from CD patients suggested that the minor allele (rs12212067) mapped to the FOXO3 locus, affects disease burden and outcome.^52^ Besides, members of the FOXO pathway, which we found to be downregulated in UC patients, are also expressed in lower levels in UC patients compared with non-IBD controls.^57^ Although it cannot be be confirmed independently using our own data alone (due to the lack of healthy/non-IBD samples in our study), the above observations suggest that the FOXO pathway could be downregulated by a bigger margin in UC than ileal CD when compared with healthy controls, which is in line with the suggested stratification of IBD using genetic data.^5^ However, we did not find any evidence from publicly available resources for a basal intestinal expression of the FOXO pathway in the non-IBD/healthy context. The above combination of evidence suggests that the modulation of the FOXO pathway could be part of a molecular phenotype associated with the pathogenesis at the primary disease site(s), although further independent validation in larger cohorts is warranted. Although it is not clear if and how the ileal phenotype affects the potential relationship between IBD disease burden and the FOXO3 mutation, the refractory nature of ileal CD^58^ as manifested across multiple clinical trials and additional meta-analysis^59 60^ suggests that novel targets are indeed required. FOXO pathway members (*BCL6, CDKN1A, BTG1, GADD45A, KLF4*) which are upregulated in ileal CD patients, could potentially fill this therapeutic gap (table 2). None of them have yet been targeted in clinical trials for IBD or the other IMIDs. BCL6 builds a compelling case as a potential novel target for ileal CD, since it modulates follicular helper T-cells, follicular regulatory T-cells^61^ in IBD, controls the inflammatory activity of regulatory T-cells,^62^ and drives the formation of the Th17 lineage of T-cells^63^ which characterise ileal CD.^6 64^ However, despite its potential disease relevance, further investigations need to be carried out to ascertain the (side)effects on other T-cell lineages when BCL6 would be targeted.

**Table 2:** Proposed targets for therapeutic targeting in UC and/or ileal CD.

Protein name	General functions	Transcription factor	UC versus L1 expression trend	UC versus non-IBD expression trend	Druggability (two sources)	Module hub?	Drugs targeting the protein (Disease–Clinical Trial ID)	ADRs
TUBB2A (Tubulin beta-2A chain)	Key mediator in the pathogenesis of PSC-IBD^65^	No	DOWN	–	No	No	Paclitaxel (Psoriasis–NCT00006276)	No
TUBB4B (Tubulin beta-4B chain)	Tubulin reorganisation^85^	No	DOWN	–	No	Yes	Paclitaxel (Psoriasis–NCT00006276)	No
TNFAIP3 (Tumour necrosis factor alpha-induced protein 3)	Master switch mediating anti-inflammatory feedback loop in CD4+T cells^86^; Modulates intestinal autophagic response and inflammatory signalling^87^	No	DOWN	–	Yes	No	–	No
GPR183 (G-protein coupled receptor 183)	Upregulated in CD4 T cells during inflammation^88^; Regulation of interferon and autophagy activity^89^; Promotes interaction of CD4 T cells with other immune cell-types^90^	No	DOWN	UP	Yes	No	–	No
PRKCQ (Protein kinase C theta type)	IBD susceptibility loci^38^; Modulation of T-cell differentiation and regulation of T-cell functions^91 92^	No	DOWN	–	Yes	No	Sotrastaurin (Ulcerative colitis –NCT00572585); Sotrastaurin (Psoriasis–CT00885196)	No
SOCS1 (Suppressor of cytokine signalling 1)	Modulates IFN-gamma and IL-4 dependent IBD pathogenesis^93^; Downregulation in T cells of patients with IBD^94^; Mediation of type-1 IFN-STAT1 signalling^94^; Regulator of intestinal immune tolerance^95^	No	DOWN	UP	Yes	No	–	No
PTGER4 (Prostaglandin E2 receptor EP4 subtype)	Significant association between PTGER4 variants and CD^38^; promotion of IFN-gamma producing Th1 and IL-17 producing Th17cells^96^	No	DOWN	–	No	No	Rivenprost (Ulcerative colitis–NCT00296556); CR-6086 (Rheumatoid arthritis–NCT03163966)	Yes
SLC2A3 (Solute carrier family 2, facilitated glucose transporter member 3)	Expressed in resting^97^ and activated CD4 T cells^98^	No	DOWN	UP	No	Yes	–	No
NR4A2 (Nuclear receptor subfamily 4 group member 2 protein)	Regulates differentiation of CD4 T cells^99^; intermediated marker for macrophage activation^100^; modulation of MIF and MAPK signalling^101^; T-cell homeostatic functions^67^	Yes	DOWN	UP	No	Yes	–	No
CXCR4 (C-X-C chemokine receptor type 4)	Participates in epithelial barrier responses^102^; Expressed in T cells on exposure to Th2 cytokines^103^; Downregulated in T cells during leucocyte removal filter (LCAP) therapy in UC patients^104^	No	DOWN	UP	Yes	No	-	No
BCL6 (B-cell lymphoma 6 protein)	T-cell proliferation in patients with IBD^61^; Mediates ATF3-depdendent protection against colitis^105^	Yes	DOWN	UP	No	No	–	No

ADR, adverse drug reaction; CD, Crohn’s disease; IBD, inflammatory bowel disease; LCAP, Leukocytapheresis; PSC, primary sclerosing cholangitis; UC, ulcerative colitis.

We also identified several already used targets which could be potentially repurposed to treat ileal CD. These include *TUBB2A*, *TUBB4B* and *PRKCQ* which are targeted in PS and *PTGER4* which is targeted in RA. *TUBB2A*, upregulated in ileal CD in our study, was also identified as a key potential mediator in the pathogenesis of PSC.^65^ Although PRKCQ and PTGER4 have been used as targets in UC, they have not yet been tested in (ileal) CD.

Beyond pathway representations, we identified regulators and regulatory mechanisms influencing pathway activities in the circulating CD4 T-cells differentiating ileal CD from UC patients. The inferred regulatory network led to the identification of key regulators such as *JUND, JUN, FOS, JUNB, KLF6*, *NR4A1, NR4A2, EGR1, KLF2* and *KLF9*. Most of these regulators, with the exception of *EGR1* and *NR4A1*, do not display any large difference in terms of transcriptional activity (as determined by the conservative criteria of differential expression) between the two patient groups, suggesting that they could be post-translationally modified and thereby change gene expression. Besides their documented roles in maintaining T cell functions,^66 67^ some of them like *EGR1, JUN* and *FOS* were also found to act in concert with the MAPK signalling pathway,^68 69^ suggesting the exertion of a concerted effect on the clinical phenotype. However, none of the TFs driving the expression of the disease location-associated genes are targeted either in IBD or the four IMIDs considered. This could be due to the intrinsic difficulty of targeting transcription factors in general which are usually localised within the nucleus. Nevertheless, the advent of novel molecules like anti-sense oligonucleotides^70^ opens up the possibility of expanding the applicability of transcription factors as drug targets for IBD. Moreover, *NR4A1* and *NR4A2* (which is both a hub gene involved in IBD pathogenesis as well as being upregulated in ileal CD vs UC) belong to the category of nuclear receptors which are a class of transcription factors known for their potential therapeutic efficacies in IBD.^71^

Despite the significance of our findings, we acknowledge several pitfalls to our study. First, we have not profiled the wide-variety of cell types which could be involved in the pathogenesis of IBD in general and rather focused on two cell types, one from each arm of the immune system. Second, we profiled gene expression from purified cell-types derived from the peripheral blood of patients with IBD. Since the primary disease site(s) were excluded for sampling, the source of the signals could not be attributed to the primary sites. Third, we did not consider other omic layers which are known to be associated with disease location-associated phenotypes. Furthermore, more granular phenotypes such as disease extent (E1, E2, E3), especially for UC patients was not incorporated into the analysis. Finally, independent validation in larger cohorts with balanced sample numbers for each of the disease subtypes (ileal CD, ileocolonic CD, colonic CD and UC) and comparison with healthy controls at the level of the investigated cell types need to be performed.

## Conclusion

In the current study, we harnessed a bioinformatic approach combining supervised and unsupervised analyses underpinning both extremes of the disease location spectrum through circulating monocytes and CD4 T cell transcriptomics. We observed that disease location in patients with IBD tend to have a more pronounced imprintation on the expression of circulating CD4 T-cells than monocytes. We report specific mechanisms including key regulators and hubs in blood derived CD4 T cell which could potentially drive the expression differences observed between ileal CD and UC patients. Our study provides an initial glimpse into the molecular differences of both entities and paves the way for novel drug targets.

## Data Availability

All data relevant to the study are included in the article or uploaded as supplementary information.
