# Supplementary material for: Distinct transcriptional signatures in purified circulating immune cells drive heterogeneity in disease location in IBD
Source: BMJ Open Gastroenterol. 2023 Feb 6;10(1):e001003. doi: 10.1136/bmjgast-2022-001003 (PMC9906185; doi:10.1136/bmjgast-2022-001003)
Supplement: Supplementary data [file bmjgast-2022-001003supp001.pdf]

**Variable**	**N**	**CD**, N = 87[1]	**UC**, N = 38[1]	**p-value**[2]
**Gender**	125			0.8
F		48 (55%)	22 (58%)	
M		39 (45%)	16 (42%)	
**Age**	125	36 (28, 49)	42 (28, 57)	0.2
**Disease.duration**	125	12 (4, 23)	7 (2, 14)	0.025
**CRP**	123	6 (3, 18)	2 (1, 8)	0.001
**Previous.antiTNF.use**	125	60 (69%)	23 (61%)	0.4
**PreviousVDZ.use**	125	32 (37%)	2 (5.3%)	<0.001
**Disease.behaviour**	87			
B1		34 (39%)	0 (NA%)	
B2		33 (38%)	0 (NA%)	
B3		20 (23%)	0 (NA%)	
**Perianal.symptoms**	87	36 (41%)	0 (NA%)	
**Steroid.use**	125			0.006
No		62 (71%)	22 (58%)	
Systemic		14 (16%)	2 (5.3%)	
Topical		11 (13%)	14 (37%)	
**Smoking.status**	125			0.033
Active-smoker		20 (23%)	2 (5.3%)	
Former-smoker		23 (26%)	16 (42%)	
Non-smoker		44 (51%)	20 (53%)	

[1] n (%); Median (IQR)
[2] Pearson's Chi-squared test; Wilcoxon rank sum test; Fisher's exact test
