# Supplementary figures and images for: Distinct transcriptional signatures in purified circulating immune cells drive heterogeneity in disease location in IBD
Source: BMJ Open Gastroenterol. 2023 Feb 6;10(1):e001003. doi: 10.1136/bmjgast-2022-001003 (PMC9906185; doi:10.1136/bmjgast-2022-001003)

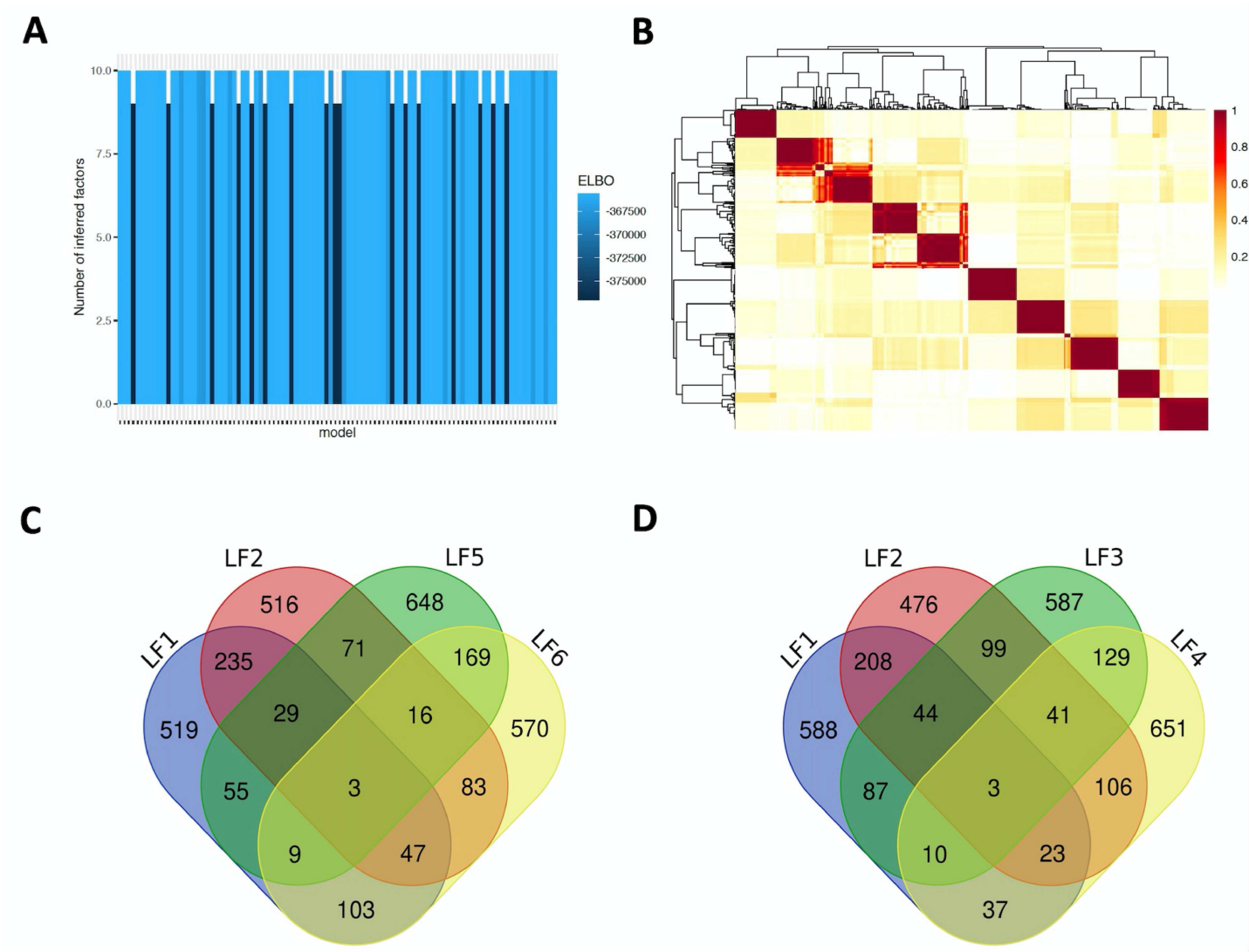

Supplement: Supplementary data [file bmjgast-2022-001003supp009.pdf]

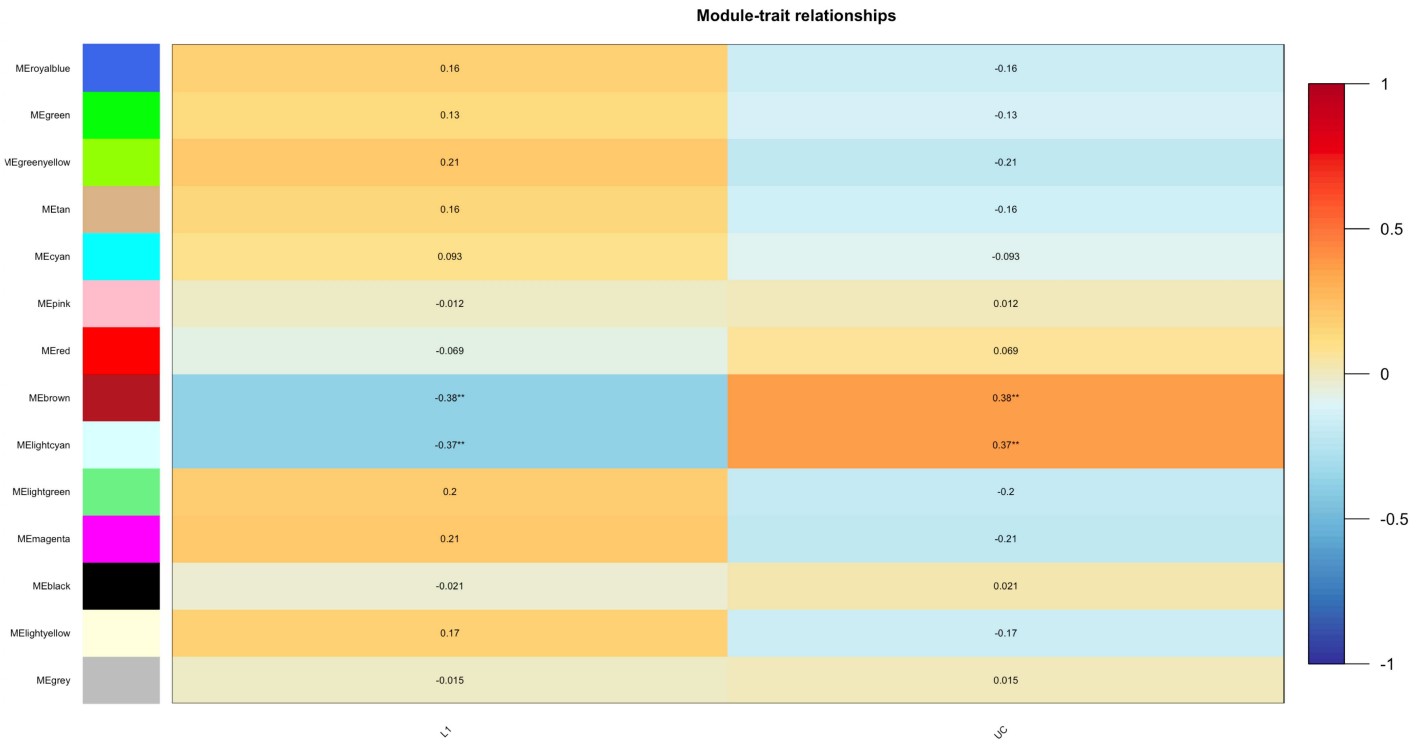

Supplement: Supplementary data [file bmjgast-2022-001003supp010.pdf]

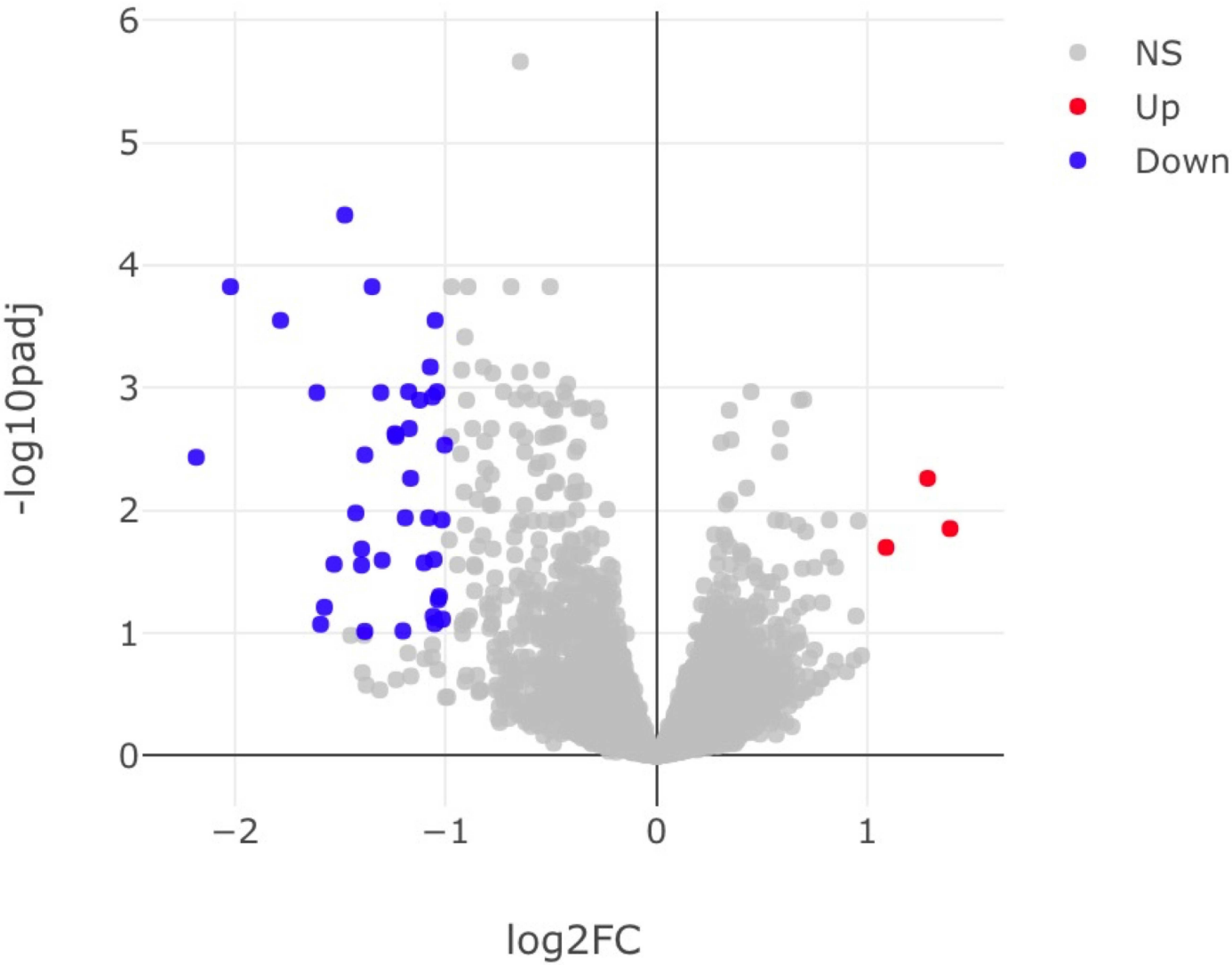

Supplement: Supplementary data [file bmjgast-2022-001003supp011.pdf]

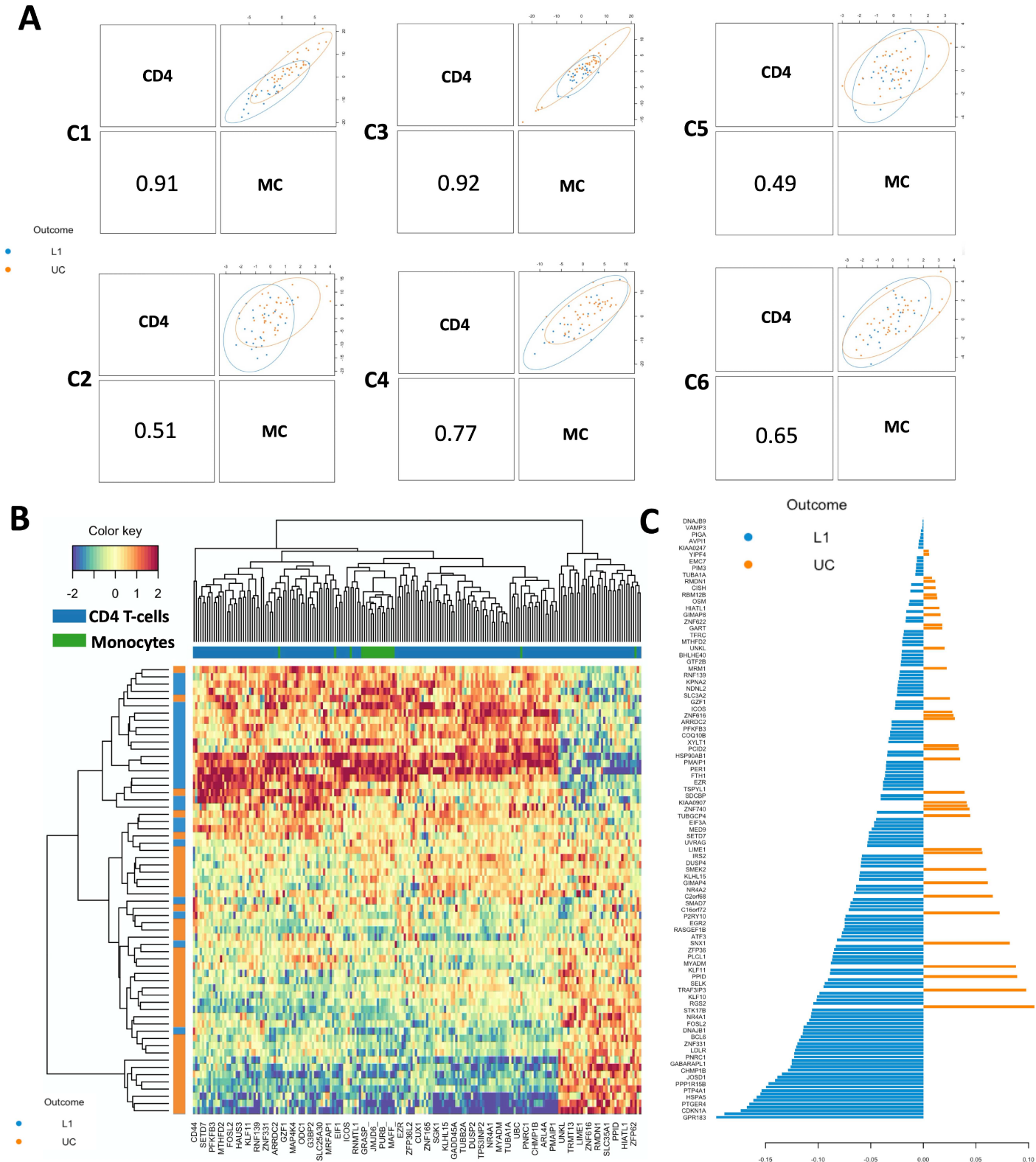

Supplement: Supplementary data [file bmjgast-2022-001003supp012.pdf]

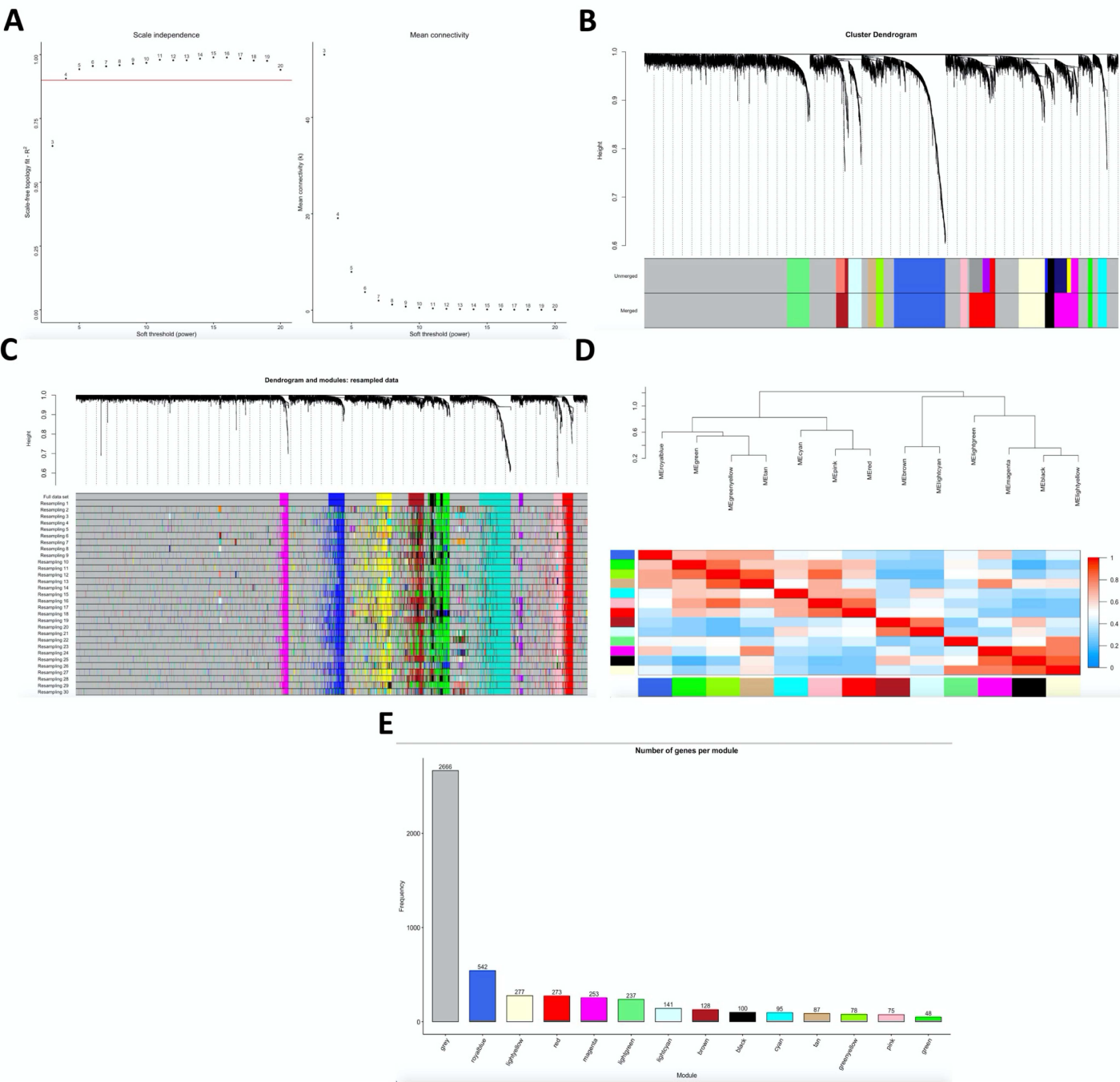

Supplement: Supplementary data [file bmjgast-2022-001003supp013.pdf]

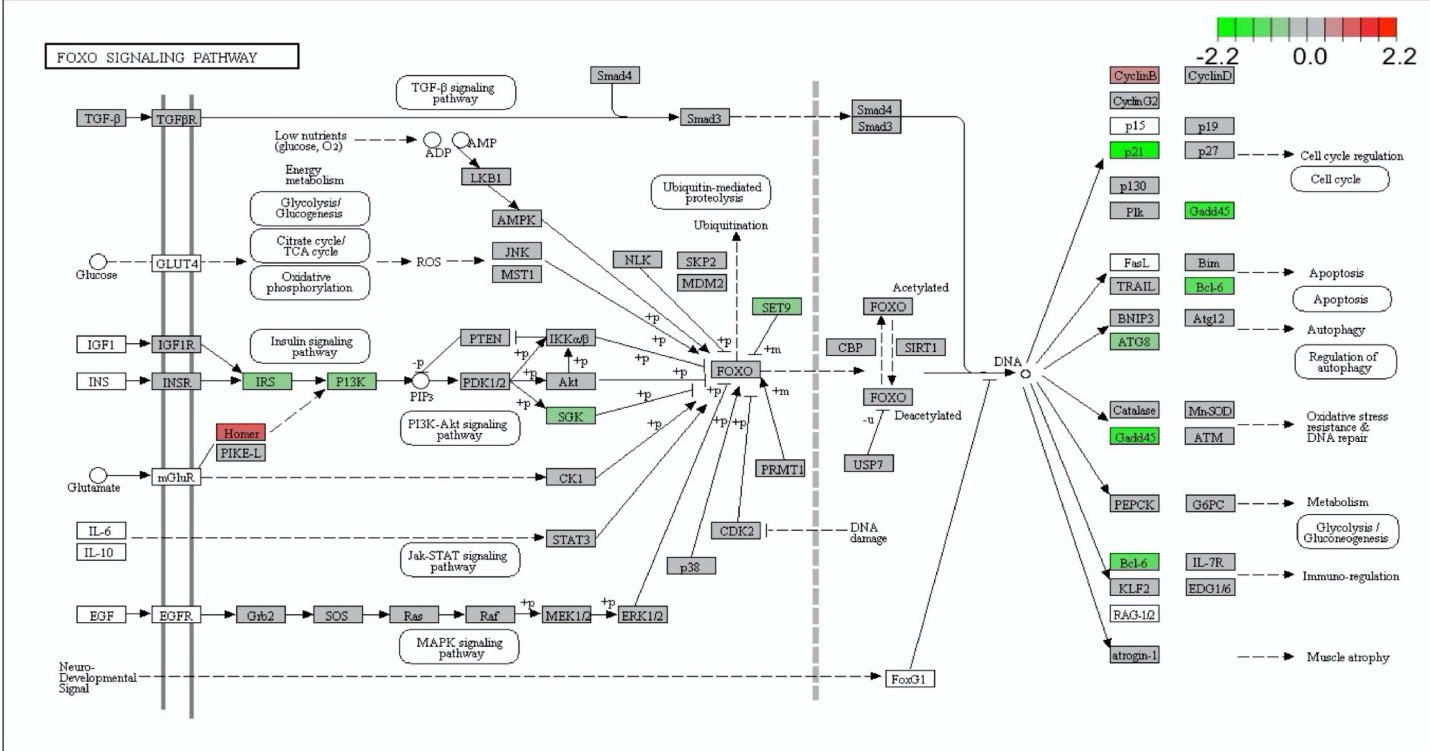

Supplement: Supplementary data [file bmjgast-2022-001003supp014.pdf]

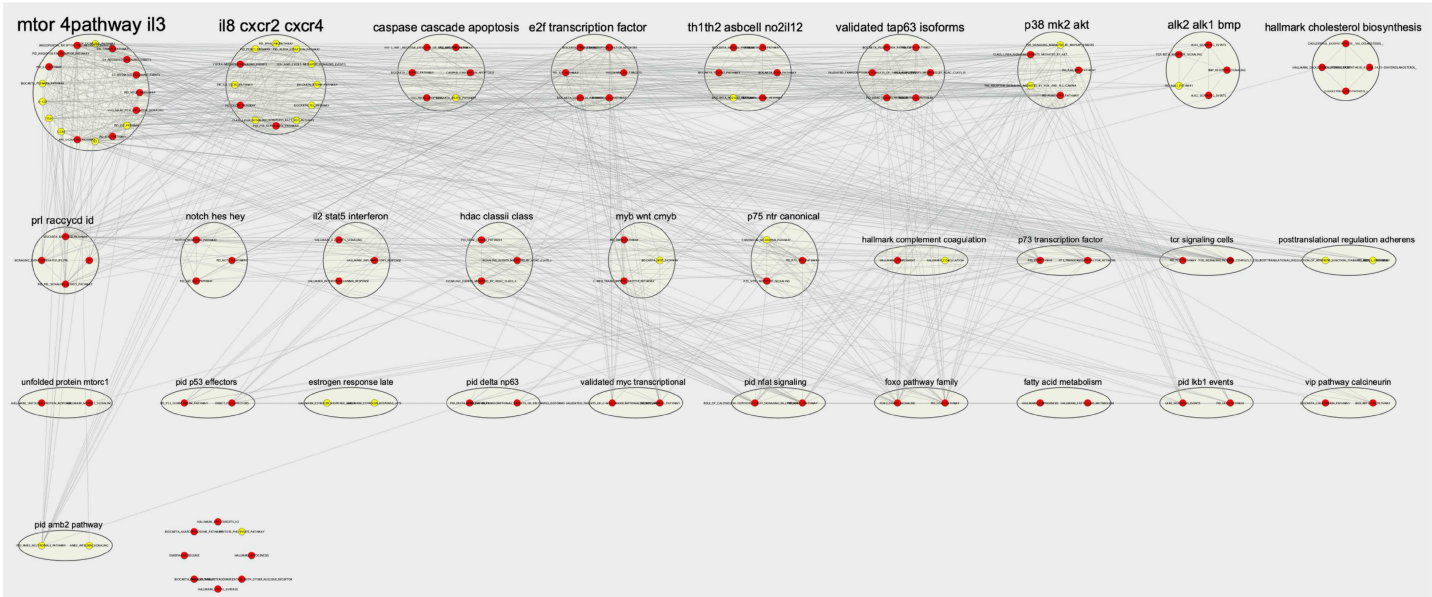

Supplement: Supplementary data [file bmjgast-2022-001003supp015.pdf]

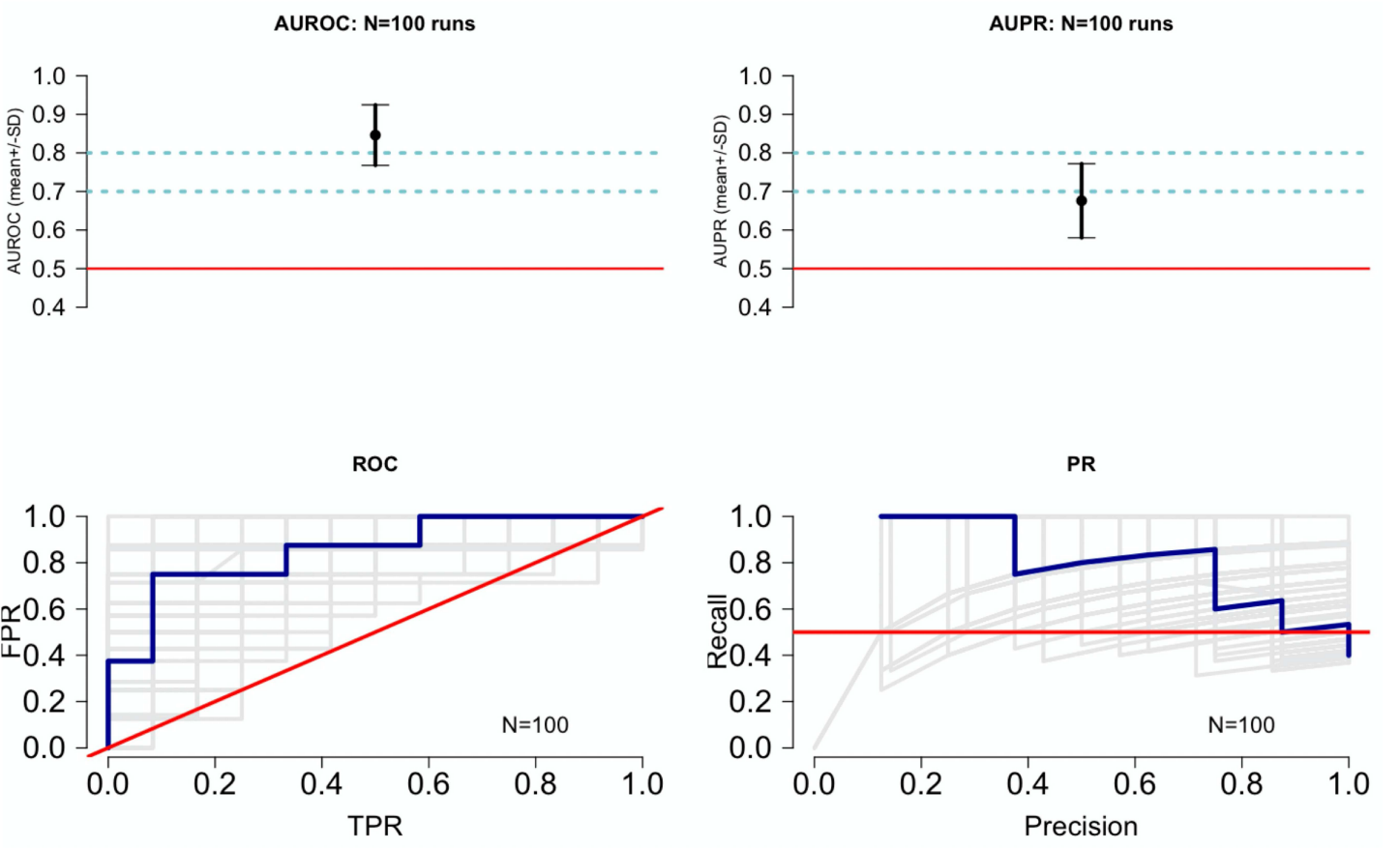

Supplement: Supplementary data [file bmjgast-2022-001003supp016.pdf]

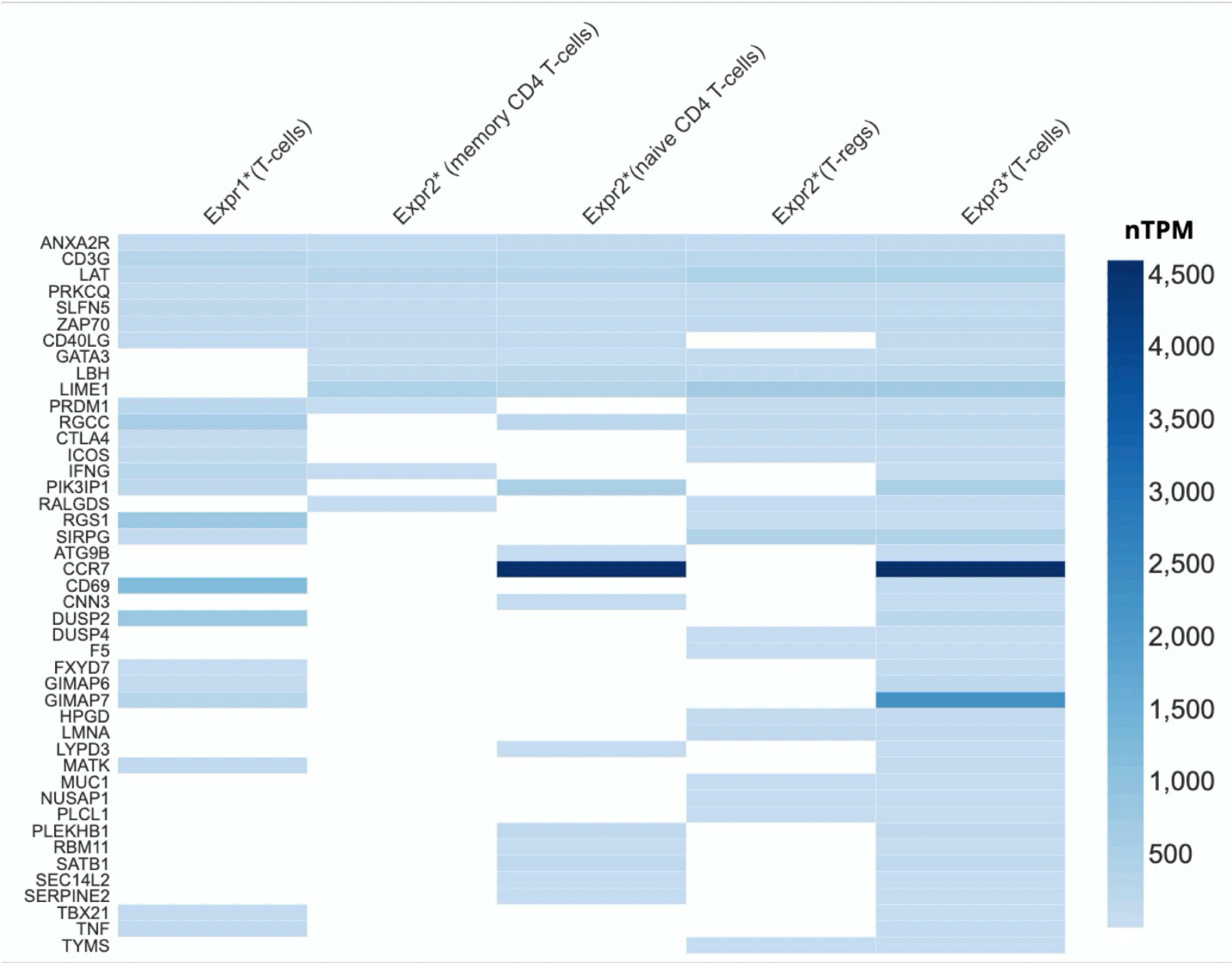

Supplement: Supplementary data [file bmjgast-2022-001003supp017.pdf]

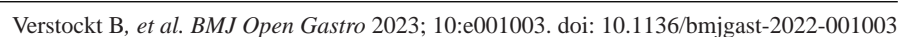

Supplement: Supplementary data [file bmjgast-2022-001003supp018.pdf]

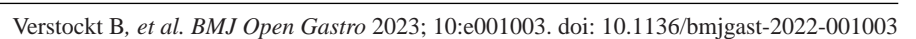

Supplement: Supplementary data [file bmjgast-2022-001003supp019.pdf]

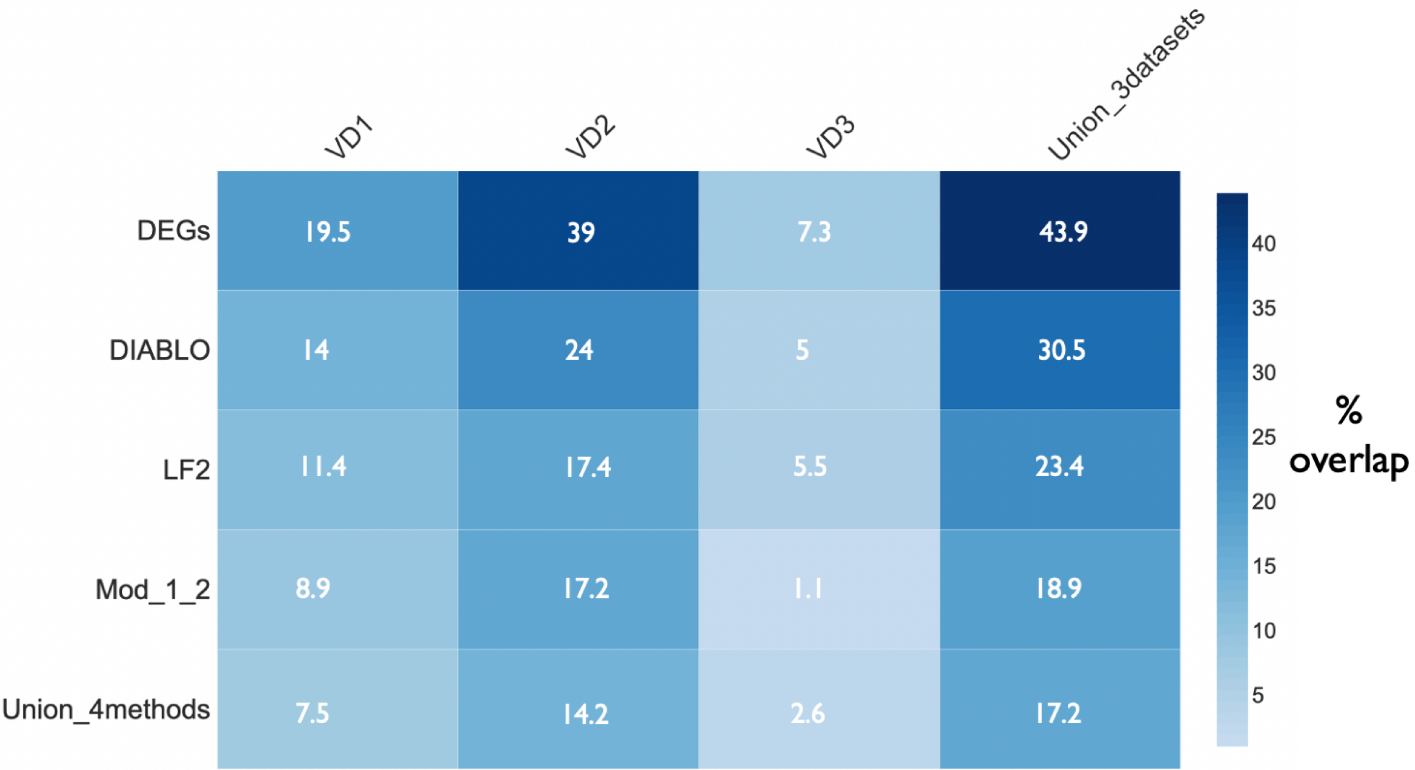

Supplement: Supplementary data [file bmjgast-2022-001003supp020.pdf]
