# Supplementary material for: Distinct transcriptional signatures in purified circulating immune cells drive heterogeneity in disease location in IBD
Source: BMJ Open Gastroenterol. 2023 Feb 6;10(1):e001003. doi: 10.1136/bmjgast-2022-001003 (PMC9906185; doi:10.1136/bmjgast-2022-001003)
Supplement: Supplementary data [file bmjgast-2022-001003supp002.pdf]

Gene	LF	Dataset	References
EMP1	LF1	CD14 transcriptomics	-
CX3CR1	LF1	CD14 transcriptomics	29743615;25024136;17285595;24625
RGCC	LF1	CD14 transcriptomics	-
NAMPTL	LF1	CD14 transcriptomics	-
NAMPT	LF1	CD14 transcriptomics	28877980;28837586;31179321
THBD	LF1	CD14 transcriptomics	23388545
NAB2	LF1	CD14 transcriptomics	-
B3GNT5	LF1	CD14 transcriptomics	-
HBEGF	LF1	CD14 transcriptomics	15749028;15300588
FOSL1	LF1	CD14 transcriptomics	23128233
PHLDA1	LF1	CD14 transcriptomics	-
HIF1A	LF1	CD14 transcriptomics	31063937;29307990;30098863
GIMAP8	LF1	CD14 transcriptomics	-
PFKFB3	LF1	CD14 transcriptomics	30541017
SLC7A5	LF1	CD14 transcriptomics	-
GPR183	LF1	CD14 transcriptomics	33511653;30742043
G0S2	LF1	CD14 transcriptomics	20848504;26339133
NRIP3	LF1	CD14 transcriptomics	-
ATP2B1	LF1	CD14 transcriptomics	17262812
SDC4	LF1	CD14 transcriptomics	30053064

Functional role or involvement in pathogenesis of IBD

-

Recruitment of immune cells to disease site; maturation of immune cells; production of infla

-

-

Upregulated in IBD; acts as a link between NAD metabolism and intestinal inflammation;

Upregulated in UC patients with neoplasia comapred to controls and UC patients without ne

-

-

Involved in IL-8 mediated signaling and cellular proliferation in the gut

IBD susceptibility locus

-

Involved in hypoxia response pathways in IBD

-

Blood transcriptional biomarker for IBD

-

SNP induced upregulation of expression in PBMCs of IBD patients; involved in intestinal path

Predictive biomarker for infliximab response in colonic CD; genetic marker for infliximab resp

-

Downregulated in UC compared to CD

Involved in epithelial cell integrity and regeneration in colitis model

ammatory cytokines; increased expression in IBD patients

oplasia

ogenesis, inflammation and development of colitis
onse
