# Supplementary material for: Distinct transcriptional signatures in purified circulating immune cells drive heterogeneity in disease location in IBD
Source: BMJ Open Gastroenterol. 2023 Feb 6;10(1):e001003. doi: 10.1136/bmjgast-2022-001003 (PMC9906185; doi:10.1136/bmjgast-2022-001003)
Supplement: Supplementary data [file bmjgast-2022-001003supp003.pdf]

Gene	LF	Dataset	References	Functional role or involvement in patho
CISH	LF1	CD4 transcrip	-	-
BCL3	LF1	CD4 transcrip	20601676; 28	IBD susceptibility locus; development of
ARID5A	LF1	CD4 transcrip	27022145	Regulates naive CD4+ T cell fate
PIM3	LF1	CD4 transcrip	22078270;30	Plays a role in CD4 T cell activation and
PER1	LF1	CD4 transcrip	28710114;30	Involved in intestinal inflammation; acts
PAQR8	LF1	CD4 transcrip	http://twas-l	Identified as a TWAS gene for CD and U
PDE4D	LF1	CD4 transcrip	30883697	Intracellular proinflammatory enzyme,
DUSP8	LF1	CD4 transcrip	-	-
PELI1	LF1	CD4 transcrip	19734906	Facilitates TRIF-dependent Toll-like rec
SLC7A5	LF1	CD4 transcrip	25701737	Involved in bacterial clearance in humar
FOSL2	LF1	CD4 transcrip	30397350	Part of a regulatory network correspon
CD200R1	LF1	CD4 transcrip	26690123	Decreased fraction of CD4+ T cells expr
WHAMM	LF1	CD4 transcrip	-	-
TNFAIP3	LF1	CD4 transcrip	29788367;33	IBD susceptibility locus; involved in resp
ST6GAL1	LF1	CD4 transcrip	-	-
FURIN	LF1	CD4 transcrip	30266770	Triggers for the Production of TGF-β by
RBM38	LF1	CD4 transcrip	-	-
PFKFB3	LF1	CD4 transcrip	27387960;22	Mediates intestinal inflammation
HIF1A	LF1	CD4 transcrip	31063937;29	Involved in hypoxia response pathways i
TRIM69	LF1	CD4 transcrip	-	-

ıgenesis of IBD

f colitis

inflammatory bowel disease; IBD predisposition
s as a clock gene whose disruption is associated with the early onset of IBD
JC
hydrolyzing and inactivating cAMP and subsequently intestinal inflammation

eptor signaling and proinflammatory cytokine production
n IECs
ding to a wound-healing program mediated by CD161+ regulatory T (Treg) cells
essing CD200R1 in IBD patients

ponse to anti-TNF treatment; master switch of cytokines such as IL-17

T Cells in the intestine

in IBD
