# Supplementary material for: Distinct transcriptional signatures in purified circulating immune cells drive heterogeneity in disease location in IBD
Source: BMJ Open Gastroenterol. 2023 Feb 6;10(1):e001003. doi: 10.1136/bmjgast-2022-001003 (PMC9906185; doi:10.1136/bmjgast-2022-001003)
Supplement: Supplementary data [file bmjgast-2022-001003supp004.pdf]

Gene	LF	Dataset	References	Functional role or involver
NR4A3	LF2	CD14 transcriptomics	-	-
IL1B	LF2	CD14 transcriptomics	32569770;28	Enhances intestinal tight ju
CD69	LF2	CD14 transcriptomics	30395204	Plays a role in immune Re
PDE4B	LF2	CD14 transcriptomics	30883697	Inhibition of PDE4 by aprer
OSM	LF2	CD14 transcriptomics	28368383;24	Drives intestinal inflamma
SNAI1	LF2	CD14 transcriptomics	-	-
EREG	LF2	CD14 transcriptomics	29129684	Regulation of intestinal ho
PTGS2	LF2	CD14 transcriptomics	16273614	Haplotype of prostaglandir
NR4A2	LF2	CD14 transcriptomics	-	-
ATF3	LF2	CD14 transcriptomics	30455690;32	Mediates a cross-regulatic
G0S2	LF2	CD14 transcriptomics	20848504;26	Predictive biomarker for ir
OLR1	LF2	CD14 transcriptomics	-	-
IL8	LF2	CD14 transcriptomics	8801200;114	Inflammatory marker in IB
NR4A1	LF2	CD14 transcriptomics	34182489;26	Susceptibility loci in famili
CD83	LF2	CD14 transcriptomics	19095953;25	Involved in the dendritic ce
MAFF	LF2	CD14 transcriptomics	-	-
CDKN1A	LF2	CD14 transcriptomics	-	-
BHLHE40	LF2	CD14 transcriptomics	29773643	Molecular switch for deter
PHLDA1	LF2	CD14 transcriptomics	34349991	The expression levels of PI
SLC7A5	LF2	CD14 transcriptomics	-	-

nent in pathogenesis of IBD

unction permeability
sponses and mucosal inflammation in IBD
nilast protected against UC, by interfering with mucosal immunity;
ition and predicts response to tumor necrosis factor-neutralizing therapy; mediates STAT3-de

omeostasis and Iintestinal stem cell regeneration
synthase 2/cyclooxygenase 2 is involved in the susceptibility to inflammatory bowel disease

on in the barrier to maintain mucosal homeostasis and immunity; important regulator of folli
nfliximab response in colonic CD; genetic marker for infliximab response

D patients: IBD pathogenesis; susceptibility gene in inflammatory bowel disease patients; pr
al CD;protective effect against inflammatory bowel disease;modulates inflammation-associa
ell pathogenesis in CD patients;Dendritic cell CD83 homotypic interactions regulate inflammat

rmining the fate of inflammatory and antiinflammatory Th1 cells.
HLDA1 in UC cases were higher in colitis, followed by dysplasia and UC-CRC, which suggested

ependent intestinal epithelial restitution;Predicts Crohn's Disease Response to Infliximab; bior

e

cular helper T (TFH) cells in gut; protects against colitis by regulating follicular helper T (TFH)

edictors of response to Vedolizumab in Inflammatory Bowel Diseases
ited intestinal fibrosis and dampens fibrogenic signaling in myofibroblasts
tion and promote mucosal homeostasis; reduced number of CD83 positive dendritic cells in CI

that this protein may be involved in the carcinogenesis of UC-CRC

narker for diagnosis; Poor Biochemical Response to Infliximab

cells in the gut;promotes intestinal epithelial cell apoptosis in Crohn's disease

) patients;Difference in Presence and Number of CD83 + Dendritic Cells in Patients with Ulcer

rative Colitis and Crohn's Disease
