# Supplementary material for: Distinct transcriptional signatures in purified circulating immune cells drive heterogeneity in disease location in IBD
Source: BMJ Open Gastroenterol. 2023 Feb 6;10(1):e001003. doi: 10.1136/bmjgast-2022-001003 (PMC9906185; doi:10.1136/bmjgast-2022-001003)
Supplement: Supplementary data [file bmjgast-2022-001003supp005.pdf]

Gene	LF	Dataset	References	Functional role or involvement in patho
NR4A2	LF2	CD4 transcrip	26564988	Protective effect against inflammatory
CSRNP1	LF2	CD4 transcrip	-	-
SIK1	LF2	CD4 transcrip	25114223	Identified as a possible drug target for c
FOSB	LF2	CD4 transcrip	-	-
NR4A1	LF2	CD4 transcrip	34182489;34	Susceptibility loci in a Japanese family v
CDKN1A	LF2	CD4 transcrip	-	-
PPP1R15A	LF2	CD4 transcrip	-	-
SLC2A3	LF2	CD4 transcrip	31618209	One among six genes whose expression
JUN	LF2	CD4 transcrip	12223450	Involved in the pathogenic mechanisms
MYADM	LF2	CD4 transcrip	-	-
MIDN	LF2	CD4 transcrip	-	-
ZFP36	LF2	CD4 transcrip	34380509	Involved in intestinal mucosal homeosta
SMAD7	LF2	CD4 transcrip	31553906;11	Controls Immunoregulatory PDL2/1-PD1
TNFAIP3	LF2	CD4 transcrip	29788367;33	IBD susceptibility locus; involved in resp
TOB2	LF2	CD4 transcrip	33247598	Over-expressed in the colonic mucosa o
FOS	LF2	CD4 transcrip	-	-
RGS1	LF2	CD4 transcrip	21795595	Involved in gut T cell trafficking
FAM46C	LF2	CD4 transcrip	-	-
PTGER4	LF2	CD4 transcrip	33558271	Prostaglandin E 2 receptor PTGER4-expr
YPEL5	LF2	CD4 transcrip	33963246	Correlation between methylation abnor

genesis of IBD
bowel disease by negatively regulating the TRAF6/TLR-IL-1R signalling axis

colitis based on small-molecule screening

with CD; modulates inflammation-associated intestinal fibrosis and dampens fibrogenic signa

levels in blood differentiated IBD patients from non-IBD controls
associated with steroid unresponsiveness in CD patients

asis
Signaling in Intestinal Inflammation and Autoimmunity;Blocking Smad7 restores TGF-beta1
onse to anti-TNF treatment; master switch of cytokines such as IL-17
f patients with UC in remission compared with the active UC and control group

ressing macrophages promote intestinal epithelial barrier regeneration upon inflammation
malities and expression patterns in CD patients

aling in myofibroblasts

signaling in chronic inflammatory bowel disease
