# Supplementary material for: Distinct transcriptional signatures in purified circulating immune cells drive heterogeneity in disease location in IBD
Source: BMJ Open Gastroenterol. 2023 Feb 6;10(1):e001003. doi: 10.1136/bmjgast-2022-001003 (PMC9906185; doi:10.1136/bmjgast-2022-001003)
Supplement: Supplementary data [file bmjgast-2022-001003supp006.pdf]

Gene	Set	IBD_lit_search	Barrier gene	AMP genes	Cell adhesio
PPP1R15A	DIABLO-LF2-mods	NA	NA	NA	NA
AMD1	LF2_mods	NA	NA	NA	NA
ATF3	DEGs-DIABLO-LF2-Mods	NA	NA	NA	NA
ATF4	LF2_mods	NA	NA	NA	NA
AVPI1	DIABLO-LF2	NA	NA	NA	NA
B3GNT2	DIABLO-LF2	1	NA	NA	NA
BBX	LF2_mods	1	NA	NA	NA
BCL6	DEGs-DIABLO-LF2	1	NA	NA	NA
BCOR	LF2_mods	NA	NA	NA	NA
BRD2	LF2_mods	NA	NA	NA	NA
BTG1	DIABLO-LF2-mods	NA	NA	NA	NA
BTG2	DIABLO-LF2-mods	1	NA	NA	NA
CD69	LF2_mods	1	NA	NA	NA
CD83	DEGs-DIABLO-LF2	1	NA	NA	NA
CDKN1A	DEGs-DIABLO-LF2-Mods	1	NA	NA	NA
CHMP1B	DIABLO-LF2-mods	NA	NA	NA	NA
CITED2	LF2_mods	NA	NA	NA	NA
CKS2	LF2_mods	NA	NA	NA	NA
CLK1	LF2_mods	NA	NA	NA	NA
CREM	DEGs-DIABLO-LF2	1	NA	NA	NA
CXCR4	LF2_mods	1	NA	NA	1
CYCS	DIABLO-LF2-mods	1	NA	NA	NA
DDIT3	LF2_mods	1	NA	NA	NA
DNAJA1	DIABLO-LF2-mods	NA	NA	NA	NA
DUSP1	LF2_mods	NA	NA	NA	NA
DUSP10	DEGs-DIABLO-LF2-Mods	NA	NA	NA	NA
DUSP2	DIABLO-LF2-mods	1	NA	NA	NA
DUSP4	DIABLO-LF2	1	NA	NA	NA
DUSP5	DIABLO-LF2	NA	NA	NA	NA
DUSP6	DIABLO-LF2	1	NA	NA	NA
DYNLL1	LF2_mods	NA	NA	NA	NA
EGR2	DEGs-DIABLO-LF2-Mods	1	NA	NA	NA
EGR3	DEGs-LF2-mods	NA	NA	NA	NA
EIF4A3	LF2_mods	NA	NA	NA	NA
EIF5	LF2_mods	1	NA	NA	NA
ERRFI1	LF2_mods	NA	NA	NA	NA
FOS	LF2_mods	1	NA	NA	NA
FOSB	LF2_mods	1	NA	NA	NA
FOSL2	DEGs-DIABLO-LF2	1	NA	NA	NA
FOXJ1	LF2_mods	1	NA	NA	NA
FZD7	LF2_mods	1	NA	NA	NA
G3BP2	DIABLO-LF2	1	NA	NA	NA
GADD45A	DIABLO-LF2-mods	NA	NA	NA	NA
GADD45B	LF2_mods	NA	NA	NA	NA
GADD45G	LF2_mods	NA	NA	NA	NA
GLA	LF2_mods	1	NA	NA	NA
GPR183	DIABLO-LF2	1	NA	NA	NA

GTF2B	DIABLO-LF2	NA		NA	NA	NA
HBEGF	DEGs-LF2		1	NA	NA	NA
HSP90AA1	LF2_mods	NA		NA	NA	NA
HSPA1B	LF2_mods	NA		NA	NA	NA
HSPA2	LF2_mods		1	NA	NA	NA
HSPA5	DIABLO-LF2		1	NA	NA	NA
HSPA8	LF2_mods	NA		NA	NA	NA
HSPH1	LF2_mods	NA		NA	NA	NA
ID1	DIABLO-LF2-mods	NA		NA	NA	NA
ID2	LF2_mods	NA		NA	NA	NA
ID3	LF2_mods	NA		NA	NA	NA
IDI1	LF2_mods	NA		NA	NA	NA
IER2	LF2_mods	NA		NA	NA	NA
IER5	LF2_mods	NA		NA	NA	NA
IFRD1	LF2_mods		1	NA	NA	NA
IRF1	DIABLO-LF2	NA		NA	NA	NA
IRF2BPL	LF2_mods	NA		NA	NA	NA
IRS2	DIABLO-LF2-mods	NA		NA	NA	NA
JOSD1	DIABLO-LF2	NA		NA	NA	NA
JUN	LF2_mods		1	NA	NA	NA
JUNB	LF2_mods	NA		NA	NA	NA
JUND	LF2_mods	NA		NA	NA	NA
KAT7	DIABLO-LF2	NA		NA	NA	NA
KBTBD8	LF2_mods	NA		NA	NA	NA
KLF10	DIABLO-LF2		1	NA	NA	NA
KLF11	DIABLO-LF2	NA		NA	NA	NA
KLF16	LF2_mods	NA		NA	NA	NA
KLF2	LF2_mods		1	NA	NA	NA
KLF4	DEGs-DIABLO-LF2		1	NA	NA	NA
KLF5	LF2_mods		1	NA	NA	NA
KLF6	LF2_mods		1	NA	NA	NA
KLF9	LF2_mods	NA		NA	NA	NA
KLHL15	DIABLO-LF2-mods	NA		NA	NA	NA
KPNA2	DIABLO-LF2-mods	NA		NA	NA	NA
LBH	LF2_mods	NA		NA	NA	NA
LDHA	DIABLO-LF2	NA		NA	NA	NA
LIMD1	DIABLO-LF2		1	NA	NA	NA
LMNA	DEGs-LF2-mods	NA		NA	NA	NA
MAFF	DIABLO-LF2	NA		NA	NA	NA
MARCKSL1	LF2_mods	NA		NA	NA	NA
MAT2A	LF2_mods	NA		NA	NA	NA
MDM4	LF2_mods	NA		NA	NA	NA
MEPCE	LF2_mods	NA		NA	NA	NA
MT-ATP6	LF2_mods	NA		NA	NA	NA
MT-CO1	LF2_mods	NA		NA	NA	NA
MT-CO2	LF2_mods	NA		NA	NA	NA
MT-CO3	LF2_mods	NA		NA	NA	NA
MT-ND1	LF2_mods	NA		NA	NA	NA

MT-ND2	LF2_mods	NA		NA	NA	NA
MT-ND3	LF2_mods	NA		NA	NA	NA
MT-ND4	LF2_mods	NA		NA	NA	NA
MT-ND4L	LF2_mods	NA		NA	NA	NA
MT-ND5	LF2_mods	NA		NA	NA	NA
MTRNR2L8	LF2_mods	NA		NA	NA	NA
MXD1	LF2_mods	NA		NA	NA	NA
MYLIP	LF2_mods		1	NA	NA	NA
NAMPT	DIABLO-LF2	NA		NA	NA	NA
NAPEPLD	DEGs-DIABLO	NA		NA	NA	NA
NEU1	LF2_mods	NA		NA	NA	NA
NFIL3	DEGs-DIABLO-LF2	NA		NA	NA	NA
NFKBIA	DIABLO-LF2-mods		1	NA	NA	NA
NR1D2	DIABLO-LF2		1	NA	NA	NA
NR4A1	DEGs-DIABLO-LF2-Mods		1	NA	NA	NA
NR4A2	DEGs-DIABLO-LF2-Mods		1	NA	NA	NA
NUFIP2	LF2_mods		1	NA	NA	NA
OAT	LF2_mods	NA		NA	NA	NA
ODC1	DIABLO-LF2	NA		NA	NA	NA
OSM	DIABLO-LF2		1	NA	NA	NA
P2RY10	DIABLO-LF2	NA		NA	NA	NA
PER1	DIABLO-LF2		1	NA	NA	NA
PFKFB3	DIABLO-LF2		1	NA	NA	NA
PIGA	DIABLO-LF2	NA		NA	NA	NA
PIM3	DIABLO-LF2		1	NA	NA	NA
PLK2	LF2_mods	NA		NA	NA	NA
PMAIP1	DIABLO-LF2-mods		1	NA	NA	NA
PNP	LF2_mods	NA		NA	NA	NA
PNRC1	DIABLO-LF2-mods	NA		NA	NA	NA
POU3F1	LF2_mods	NA		NA	NA	NA
PRKCQ	DIABLO-LF2		1	NA	NA	NA
PTGER4	DIABLO-LF2-mods		1	NA	NA	NA
PTP4A1	DIABLO-LF2-mods	NA		NA	NA	NA
RAB33B	LF2_mods		1	NA	NA	NA
RNF139	DIABLO-LF2-mods	NA		NA	NA	NA
RNF19B	DIABLO-LF2-mods	NA		NA	NA	NA
SERTAD1	LF2_mods	NA		NA	NA	NA
SGK1	DIABLO-LF2-mods	NA		NA	NA	NA
SGMS1	DIABLO-LF2	NA		NA	NA	NA
SIAH2	DIABLO-LF2	NA		NA	NA	NA
SIK1	DEGs-DIABLO-LF2-Mods	NA		NA	NA	NA
SIRT1	LF2_mods	NA		NA	NA	NA
SLC1A5	LF2_mods		1	NA	NA	NA
SLC2A3	DIABLO-LF2-mods	NA		NA	NA	NA
SLC30A1	LF2_mods		1	NA	NA	NA
SLC35A1	DIABLO-LF2	NA		NA	NA	NA
SLC35A2	LF2_mods	NA		NA	NA	NA
SLC38A2	LF2_mods	NA		NA	NA	NA

SLC3A2	DIABLO-LF2	NA		NA	NA	NA
SMAD7	DEGs-DIABLO-LF2-Mods	NA		NA	NA	NA
SOCS1	LF2_mods		1	NA	NA	NA
SOCS3	LF2_mods		1	NA	NA	NA
SPTY2D1	DIABLO-LF2-mods		1	NA	NA	NA
SRGN	DIABLO-LF2		1	NA	NA	NA
TAF13	DIABLO-LF2	NA		NA	NA	NA
TFRC	DIABLO-LF2	NA		NA	NA	NA
TIPARP	DIABLO-LF2-mods		1	NA	NA	NA
TNFAIP3	DEGs-DIABLO-LF2-Mods		1	NA	NA	NA
TP53INP2	DEGs-DIABLO-LF2-Mods	NA		NA	NA	NA
TSC22D2	LF2_mods		1	NA	NA	NA
TUBA1A	DIABLO-LF2-mods	NA		NA	NA	NA
TUBA1B	LF2_mods	NA		NA	NA	NA
TUBA4A	LF2_mods	NA		NA	NA	NA
TUBB2A	DEGs-DIABLO-LF2-Mods	NA		NA	NA	NA
TUBB4B	LF2_mods	NA		NA	NA	NA
UBC	DIABLO-LF2		1	NA	1	NA
UBE2S	LF2_mods	NA		NA	NA	NA
XYLT1	DIABLO-LF2	NA		NA	NA	NA
YPEL5	DIABLO-LF2-mods	NA		NA	NA	NA
YRDC	DIABLO-LF2	NA		NA	NA	NA
ZFP36	DIABLO-LF2-mods	NA		NA	NA	NA
ZFP36L2	DIABLO-LF2-mods	NA		NA	NA	NA
ZNF331	DEGs-DIABLO-LF2		1	NA	NA	NA
ZNF622	DIABLO-LF2	NA		NA	NA	NA
ZNF703	LF2_mods	NA		NA	NA	NA

IBD suscepti	eQTL	CD_OT_drug	UC_OT_drug	IBD_OT_dru	PS_OT_drug	SC_OT_drug
NA	NA	NA	NA	NA	NA	NA
NA	NA	NA	NA	NA	NA	NA
NA	NA	NA	NA	NA	NA	NA
NA	NA	NA	NA	NA	NA	NA
NA	NA	NA	NA	NA	NA	NA
1	NA	NA	NA	NA	NA	NA
NA	1	NA	NA	NA	NA	NA
NA	NA	NA	NA	NA	NA	NA
NA	NA	NA	NA	NA	NA	NA
NA	NA	NA	NA	NA	NA	NA
NA	NA	NA	NA	NA	NA	NA
NA	NA	NA	NA	NA	NA	NA
NA	NA	NA	NA	NA	NA	NA
NA	1	NA	NA	NA	NA	NA
NA	NA	NA	NA	NA	NA	NA
NA	NA	NA	NA	NA	NA	NA
NA	NA	NA	NA	NA	NA	NA
NA	NA	NA	NA	NA	NA	NA
NA	NA	NA	NA	NA	NA	NA
1	NA	NA	NA	NA	NA	NA
NA	NA	NA	NA	1	NA	NA
NA	1	NA	NA	NA	NA	NA
NA	NA	NA	NA	NA	NA	NA
NA	NA	NA	NA	NA	NA	NA
NA	NA	NA	NA	NA	NA	NA
NA	NA	NA	NA	NA	NA	NA
NA	NA	NA	NA	NA	NA	NA
NA	NA	NA	NA	NA	NA	NA
NA	NA	NA	NA	NA	NA	NA
NA	NA	NA	NA	NA	NA	NA
NA	NA	NA	NA	NA	NA	NA
NA	NA	NA	NA	NA	NA	NA
NA	NA	NA	NA	NA	NA	NA
NA	NA	NA	NA	NA	NA	NA
NA	1	NA	NA	NA	NA	NA
NA	NA	NA	NA	NA	NA	NA
1	NA	NA	NA	NA	NA	NA
NA	NA	NA	NA	NA	NA	NA
1	1	NA	NA	NA	NA	NA
NA	NA	NA	NA	NA	NA	NA
NA	NA	NA	NA	NA	NA	NA
NA	1	NA	NA	NA	NA	NA
NA	NA	NA	NA	NA	NA	NA
NA	NA	NA	NA	NA	NA	NA
NA	NA	NA	NA	NA	NA	NA
NA	NA	NA	NA	NA	NA	NA
1	NA	NA	NA	NA	NA	NA

NA	NA	NA	NA	NA	NA	NA
NA	NA	NA	NA	NA	NA	NA
NA	NA	NA	NA	NA	NA	NA
NA	NA	NA	NA	NA	NA	NA
NA	NA	NA	NA	NA	NA	NA
NA	NA	NA	NA	NA	NA	NA
NA	NA	NA	NA	NA	NA	NA
NA	NA	NA	NA	NA	NA	NA
NA	NA	NA	NA	NA	NA	NA
NA	NA	NA	NA	NA	NA	NA
NA	NA	NA	NA	NA	NA	NA
NA	NA	NA	NA	NA	NA	NA
NA	NA	NA	NA	NA	NA	NA
NA	NA	NA	NA	NA	NA	NA
NA	1	NA	NA	NA	NA	NA
NA	NA	NA	NA	NA	NA	NA
NA	NA	NA	NA	NA	NA	NA
NA	NA	NA	NA	NA	NA	NA
NA	NA	NA	NA	NA	NA	NA
NA	NA	NA	NA	NA	NA	NA
NA	NA	NA	NA	NA	NA	NA
NA	NA	NA	NA	NA	NA	NA
NA	NA	NA	NA	NA	NA	NA
NA	NA	NA	NA	NA	NA	NA
NA	NA	NA	NA	NA	NA	NA
NA	NA	NA	NA	NA	NA	NA
NA	NA	NA	NA	NA	NA	NA
NA	1	NA	NA	NA	NA	NA
NA	NA	NA	NA	NA	NA	NA
NA	1	NA	NA	NA	NA	NA
NA	NA	NA	NA	NA	NA	NA
NA	NA	NA	NA	NA	NA	NA
NA	NA	NA	NA	NA	NA	NA
NA	NA	NA	NA	NA	NA	NA
NA	NA	NA	NA	NA	NA	NA
NA	NA	NA	NA	NA	NA	NA
NA	1	NA	NA	NA	NA	NA
NA	NA	NA	NA	NA	NA	NA
NA	NA	NA	NA	NA	NA	NA
NA	NA	NA	NA	NA	NA	NA
NA	NA	NA	NA	NA	NA	NA
NA	NA	NA	NA	NA	NA	NA
NA	NA	NA	NA	NA	NA	NA
NA	NA	NA	NA	NA	NA	NA
NA	NA	NA	NA	NA	NA	NA
NA	NA	NA	NA	NA	NA	NA
NA	NA	NA	NA	NA	NA	NA
NA	NA	NA	1	1	1	NA

NA	NA	NA	1	1	1	NA
NA	NA	NA	1	1	1	NA
NA	NA	NA	1	1	1	NA
NA	NA	NA	1	1	1	NA
NA	NA	NA	1	1	1	NA
NA	NA	NA	NA	NA	NA	NA
NA	NA	NA	NA	NA	NA	NA
NA	1	NA	NA	NA	NA	NA
NA	NA	NA	NA	NA	NA	NA
NA	NA	NA	NA	NA	NA	NA
NA	NA	NA	NA	NA	NA	NA
NA	NA	NA	NA	NA	NA	NA
NA	NA	NA	NA	NA	NA	NA
NA	1	NA	NA	NA	NA	NA
NA	NA	NA	NA	NA	NA	NA
NA	NA	NA	NA	NA	NA	NA
NA	1	NA	NA	NA	NA	NA
NA	NA	NA	NA	NA	NA	NA
NA	NA	NA	NA	NA	NA	NA
NA	NA	NA	NA	NA	NA	NA
NA	NA	NA	NA	NA	NA	NA
NA	NA	NA	NA	NA	NA	NA
NA	NA	NA	NA	NA	NA	NA
NA	NA	NA	NA	NA	NA	NA
NA	NA	NA	NA	NA	NA	NA
NA	NA	NA	NA	NA	NA	NA
NA	1	NA	NA	NA	NA	NA
NA	NA	NA	NA	NA	NA	NA
NA	NA	NA	NA	NA	NA	NA
NA	NA	NA	NA	NA	NA	NA
1	NA	NA	1	1	1	NA
1	NA	NA	1	1	NA	NA
NA	NA	NA	NA	NA	NA	NA
NA	1	NA	NA	NA	NA	NA
NA	NA	NA	NA	NA	NA	NA
NA	NA	NA	NA	NA	NA	NA
NA	NA	NA	NA	NA	NA	NA
NA	NA	NA	NA	NA	NA	NA
NA	NA	NA	NA	NA	NA	NA
NA	NA	NA	NA	NA	NA	NA
NA	NA	NA	NA	NA	NA	NA
NA	NA	NA	1	1	1	NA
NA	NA	NA	NA	NA	NA	NA
NA	NA	NA	NA	NA	NA	NA
NA	1	NA	NA	NA	NA	NA
NA	NA	NA	NA	NA	NA	NA
NA	NA	NA	NA	NA	NA	NA
NA	NA	NA	NA	NA	NA	NA

NA	NA	NA	NA	NA	NA	NA
NA	NA	1	1	1	NA	NA
1	NA	NA	NA	NA	NA	NA
NA	NA	NA	NA	NA	NA	NA
NA	1	NA	NA	NA	NA	NA
NA	NA	NA	NA	NA	NA	NA
NA	NA	NA	NA	NA	NA	NA
NA	NA	NA	NA	NA	NA	NA
NA	1	NA	NA	NA	NA	NA
1	NA	NA	NA	NA	NA	NA
NA	NA	NA	NA	NA	NA	NA
NA	1	NA	NA	NA	NA	NA
NA	NA	NA	NA	NA	NA	NA
NA	NA	NA	NA	NA	NA	NA
NA	NA	NA	NA	NA	NA	NA
NA	NA	NA	NA	NA	1	NA
NA	NA	NA	NA	NA	1	NA
NA	NA	NA	NA	NA	NA	NA
NA	NA	NA	NA	NA	NA	NA
NA	NA	NA	NA	NA	NA	NA
NA	NA	NA	NA	NA	NA	NA
NA	NA	NA	NA	NA	NA	NA
NA	NA	NA	NA	NA	NA	NA
NA	NA	NA	NA	NA	NA	NA
NA	1	NA	NA	NA	NA	NA
NA	NA	NA	NA	NA	NA	NA
NA	NA	NA	NA	NA	NA	NA

AS_OT_drug	RA_OT_drug	Druggability_1s	Druggability_2s	ADRs_TARDI	DEG_FDR_0.1_log2FC_1
NA	NA	1	NA	NA	NA
NA	NA	1	NA	NA	NA
NA	NA	1	NA	NA	1
NA	NA	1	NA	NA	NA
NA	NA	1	NA	NA	NA
NA	NA	1	NA	NA	NA
NA	NA	1	NA	NA	NA
NA	NA	1	NA	NA	1
NA	NA	1	NA	NA	NA
NA	NA	1	NA	NA	NA
NA	NA	1	NA	NA	NA
NA	NA	1	NA	NA	NA
NA	NA	1	NA	NA	NA
NA	NA	1	NA	NA	1
NA	NA	1	NA	NA	1
NA	NA	1	NA	NA	NA
NA	NA	1	NA	NA	NA
NA	NA	1	NA	NA	NA
NA	NA	1	NA	NA	NA
NA	NA	1	NA	NA	1
NA	NA	1	1	NA	NA
NA	NA	NA	NA	1	NA
NA	NA	1	NA	NA	NA
NA	NA	1	NA	NA	NA
NA	NA	1	NA	NA	NA
NA	NA	1	NA	NA	1
NA	NA	1	NA	NA	NA
NA	NA	1	NA	NA	NA
NA	NA	1	NA	NA	NA
NA	NA	1	NA	NA	NA
NA	NA	1	NA	NA	NA
NA	NA	1	NA	NA	1
NA	NA	1	NA	NA	1
NA	NA	1	NA	NA	NA
NA	NA	NA	NA	NA	NA
NA	NA	1	NA	NA	NA
NA	NA	1	NA	1	NA
NA	NA	1	NA	NA	NA
NA	NA	1	NA	NA	1
NA	NA	1	NA	NA	NA
NA	NA	1	NA	NA	NA
NA	NA	NA	NA	NA	NA
NA	NA	1	NA	NA	NA
NA	NA	1	NA	NA	NA
NA	NA	1	NA	NA	NA
NA	NA	1	NA	NA	NA
NA	NA	1	1	NA	NA

NA	NA	1	NA	NA	NA
NA	NA	1	NA	NA	1
NA	NA	1	NA	NA	NA
NA	NA	1	NA	NA	NA
NA	NA	1	NA	NA	NA
NA	NA	1	NA	NA	NA
NA	NA	1	NA	NA	NA
NA	NA	1	NA	NA	NA
NA	NA	1	NA	NA	NA
NA	NA	1	NA	NA	NA
NA	NA	1	NA	NA	NA
NA	NA	1	NA	NA	NA
NA	NA	1	NA	NA	NA
NA	NA	1	NA	NA	NA
NA	NA	NA	NA	NA	NA
NA	NA	1	NA	NA	NA
NA	NA	1	NA	NA	NA
NA	NA	1	NA	NA	NA
NA	NA	1	NA	NA	NA
NA	NA	1	NA	NA	NA
NA	NA	1	NA	NA	NA
NA	NA	1	NA	NA	NA
NA	NA	1	NA	NA	NA
NA	NA	1	NA	NA	NA
NA	NA	1	NA	NA	NA
NA	NA	1	NA	NA	NA
NA	NA	1	NA	NA	NA
NA	NA	1	NA	NA	NA
NA	NA	1	NA	NA	1
NA	NA	1	NA	NA	NA
NA	NA	1	NA	NA	NA
NA	NA	1	NA	NA	NA
NA	NA	1	NA	NA	NA
NA	NA	1	NA	NA	NA
NA	NA	1	NA	NA	NA
NA	NA	1	NA	NA	NA
NA	NA	1	NA	NA	NA
NA	NA	1	NA	1	1
NA	NA	1	NA	NA	NA
NA	NA	1	NA	NA	NA
NA	NA	1	NA	NA	NA
NA	NA	1	NA	NA	NA
NA	NA	1	NA	NA	NA
NA	NA	1	NA	NA	NA
NA	NA	1	NA	NA	NA
NA	NA	1	NA	NA	NA
NA	NA	1	NA	NA	NA
NA	1	1	1	NA	NA

NA	1	1	1	NA	NA
NA	1	1	1	NA	NA
NA	1	1	1	NA	NA
NA	1	1	1	NA	NA
NA	1	1	1	NA	NA
NA	NA	1	NA	NA	NA
NA	NA	1	NA	NA	NA
NA	NA	1	NA	NA	NA
NA	NA	1	NA	NA	NA
NA	NA	1	NA	NA	1
NA	NA	1	NA	NA	NA
NA	NA	1	NA	NA	1
NA	NA	1	NA	NA	NA
NA	NA	1	1	NA	NA
NA	NA	1	NA	NA	1
NA	NA	1	NA	NA	1
NA	NA	NA	NA	NA	NA
NA	NA	1	NA	NA	NA
NA	NA	1	NA	1	NA
NA	NA	1	NA	NA	NA
NA	NA	1	NA	NA	NA
NA	NA	1	NA	NA	NA
NA	NA	1	NA	NA	NA
NA	NA	1	NA	NA	NA
NA	NA	1	NA	NA	NA
NA	NA	1	NA	NA	NA
NA	NA	1	NA	NA	NA
NA	NA	1	NA	NA	NA
NA	NA	1	NA	NA	NA
NA	NA	1	NA	NA	NA
NA	NA	1	1	NA	NA
NA	1	1	1	1	NA
NA	NA	1	NA	NA	NA
NA	NA	1	NA	NA	NA
NA	NA	1	NA	NA	NA
NA	NA	1	NA	NA	NA
NA	NA	1	NA	NA	NA
NA	NA	1	NA	NA	NA
NA	NA	1	NA	NA	NA
NA	NA	1	NA	NA	NA
NA	NA	1	NA	NA	1
NA	NA	1	1	1	NA
NA	NA	1	NA	NA	NA
NA	NA	1	NA	NA	NA
NA	NA	1	1	NA	NA
NA	NA	1	NA	NA	NA
NA	NA	1	NA	NA	NA
NA	NA	1	NA	NA	NA

NA	NA	1	NA	NA	NA
NA	NA	1	NA	NA	1
NA	NA	1	1	NA	NA
NA	NA	1	NA	NA	NA
NA	NA	NA	NA	NA	NA
NA	NA	1	NA	NA	NA
NA	NA	1	NA	NA	NA
NA	NA	1	NA	NA	NA
NA	NA	1	NA	NA	NA
NA	NA	1	1	NA	1
NA	NA	1	NA	NA	1
NA	NA	NA	NA	NA	NA
NA	NA	1	NA	NA	NA
NA	NA	1	NA	NA	NA
NA	NA	1	NA	NA	NA
NA	1	1	NA	NA	1
NA	1	1	NA	NA	NA
NA	NA	1	NA	NA	NA
NA	NA	1	NA	NA	NA
NA	NA	1	NA	NA	NA
NA	NA	1	NA	NA	NA
NA	NA	1	NA	NA	NA
NA	NA	1	NA	NA	NA
NA	NA	1	NA	NA	NA
NA	NA	1	NA	NA	1
NA	NA	1	NA	NA	NA
NA	NA	1	NA	NA	NA

DEG_FDR_0.1_log2FC_0.5	UC vs L1 (UP/DOWN-regulation)	log2FC	IBD_path
1	DOWN	-0,8645643	0
NA	DOWN	-0,3232243	0
1	DOWN	-1,3986805	0
NA	DOWN	-0,2443773	0
1	DOWN	-0,9052901	0
NA	DOWN	-0,4722703	1
NA	DOWN	-0,2215873	1
1	DOWN	-1,1227267	0
NA	UP	0,05587804	0
NA	DOWN	-0,2257226	0
NA	DOWN	-0,365957	0
1	DOWN	-0,6636222	0
1	DOWN	-0,5867451	0
1	DOWN	-1,4781731	1
1	DOWN	-2,020562	0
1	DOWN	-0,5454792	0
NA	DOWN	-0,1403614	0
NA	DOWN	-0,4168107	0
NA	DOWN	-0,2647741	0
1	DOWN	-1,0725734	1
NA	DOWN	-0,4232301	1
1	DOWN	-0,5241672	1
NA	DOWN	-0,5505859	0
1	DOWN	-0,5837984	0
NA	DOWN	-0,4177612	0
1	DOWN	-1,0496186	0
1	DOWN	-0,7890283	0
1	DOWN	-0,9120287	0
1	DOWN	-0,814372	0
1	DOWN	-0,9827876	0
NA	DOWN	-0,1475331	0
1	DOWN	-1,3983796	0
1	DOWN	-1,3832812	0
NA	DOWN	-0,4309784	0
NA	DOWN	-0,255991	1
NA	DOWN	-0,732489	0
NA	DOWN	-0,5808991	1
NA	DOWN	-0,633634	0
1	DOWN	-1,3073953	1
NA	DOWN	-0,6915638	0
NA	DOWN	-0,5652356	0
NA	DOWN	-0,4204348	1
1	DOWN	-0,7990049	0
NA	DOWN	-0,3284407	0
NA	DOWN	-0,5295784	0
1	DOWN	-0,6166111	0
1	DOWN	-0,6457591	1

NA	DOWN	-0,4498437	0
1	DOWN	-1,3008274	0
NA	DOWN	-0,2821408	0
NA	DOWN	-0,6758139	0
NA	DOWN	-0,6488319	0
1	DOWN	-0,5040753	0
NA	DOWN	-0,1630578	0
NA	DOWN	-0,1849143	0
NA	DOWN	-1,0623889	0
NA	DOWN	-0,4185714	0
NA	UP	0,12756149	0
NA	DOWN	-0,4502897	0
NA	DOWN	-0,2811757	0
NA	DOWN	-0,4969255	0
1	DOWN	-0,5603043	1
NA	DOWN	-0,4094846	0
NA	DOWN	-0,3052449	0
1	DOWN	-0,7655747	0
NA	DOWN	-0,4794424	0
NA	DOWN	-0,5013862	0
NA	DOWN	-0,4705435	0
1	DOWN	-0,628667	0
NA	DOWN	-0,2839779	0
NA	DOWN	-0,222066	0
1	DOWN	-0,9007233	0
1	DOWN	-0,6244434	0
1	DOWN	-0,6137376	0
NA	DOWN	-0,3069521	1
1	DOWN	-1,7831689	0
1	DOWN	-0,6679578	1
1	DOWN	-0,6567398	0
NA	DOWN	-0,3599633	0
1	DOWN	-0,5337514	0
NA	DOWN	-0,7150591	0
NA	UP	0,11262509	0
NA	DOWN	-0,4290521	0
NA	UP	0,34663494	1
1	DOWN	-1,1912799	0
1	DOWN	-0,6427097	0
NA	DOWN	-0,2295067	0
NA	UP	0,07272971	0
NA	UP	0,21855993	0
NA	DOWN	-0,4049286	0
NA	UP	0,09486707	0
NA	UP	0,07064231	0
NA	UP	0,0275914	0
NA	UP	0,10709593	0
NA	UP	0,09074788	0

NA	UP	0,08623284	0
NA	UP	0,08682953	0
NA	UP	0,2015388	0
NA	UP	0,16463293	0
NA	UP	0,00445984	0
NA	DOWN	-0,0303424	0
NA	DOWN	-0,4878941	0
NA	DOWN	-0,3263268	1
1	DOWN	-0,9717678	0
1	UP	1,08974958	0
1	DOWN	-0,7825998	0
1	DOWN	-1,0548635	0
1	DOWN	-0,6581822	0
NA	DOWN	-0,3853246	1
1	DOWN	-1,3830236	0
1	DOWN	-1,0348639	0
NA	DOWN	-0,1022137	1
NA	DOWN	-0,3016626	0
NA	DOWN	-0,4655042	0
1	DOWN	-0,7751732	0
1	DOWN	-0,5132971	0
1	DOWN	-0,7722103	0
1	DOWN	-0,8207638	0
NA	DOWN	-0,4861513	0
1	DOWN	-0,6590407	0
NA	DOWN	-0,2747099	0
NA	DOWN	-0,7362575	1
NA	DOWN	-0,1402062	0
NA	DOWN	-0,3508827	0
NA	DOWN	-0,4197085	0
NA	DOWN	-0,2663772	1
1	DOWN	-0,9236738	1
1	DOWN	-0,6480574	0
NA	DOWN	-0,3062933	1
NA	DOWN	-0,3742072	0
1	DOWN	-0,6222355	0
NA	DOWN	-0,3395818	0
1	DOWN	-0,8478855	0
NA	DOWN	-0,4078453	0
NA	DOWN	-0,4704996	0
1	DOWN	-1,0293169	0
NA	DOWN	-0,3122223	0
NA	DOWN	-0,6410879	0
1	DOWN	-0,9740432	0
NA	DOWN	-0,258694	1
1	UP	0,82058971	0
NA	DOWN	-0,1651374	0
NA	DOWN	-0,3272159	0

NA	DOWN	-0,2602121	0
1	DOWN	-1,1660793	0
NA	DOWN	-0,2524845	1
1	DOWN	-0,5480009	0
1	DOWN	-0,5595209	1
1	DOWN	-0,7769913	0
1	DOWN	-0,6716295	0
NA	DOWN	-0,3775501	0
1	DOWN	-0,571003	1
1	DOWN	-1,0621379	1
1	DOWN	-1,2359345	0
1	DOWN	-0,5351233	1
NA	DOWN	-0,4667487	0
NA	DOWN	-0,1783176	0
NA	DOWN	-0,2692345	0
1	DOWN	-1,0047516	0
NA	DOWN	-0,4198666	0
NA	DOWN	-0,3728648	1
NA	DOWN	-0,578054	0
NA	DOWN	-0,3667091	0
1	DOWN	-0,7822234	0
NA	DOWN	-0,3761356	0
1	DOWN	-0,9274354	0
NA	DOWN	-0,3724031	0
1	DOWN	-1,1756283	1
NA	DOWN	-0,322366	0
NA	DOWN	-0,8311596	0

IBD_DT	nonIBD_DT	UC_meta_ar	UC_meta_ar	UC_meta_ar	hubinfo
0	0	NA	NA	NA	mod1_hub1
0	0	NA	NA	NA	NA
0	0	NA	NA	NA	NA
0	0	NA	NA	NA	NA
0	0	NA	NA	NA	NA
0	0	NA	NA	NA	NA
0	0	NA	NA	NA	NA
0	0	1	0,91164884	8	NA
0	0	NA	NA	NA	NA
0	0	NA	NA	NA	mod2_hub7
0	0	NA	NA	NA	NA
0	0	NA	NA	NA	NA
0	0	NA	NA	NA	NA
0	0	NA	NA	NA	NA
0	0	NA	NA	NA	NA
0	0	NA	NA	NA	NA
0	0	NA	NA	NA	NA
0	0	NA	NA	NA	NA
0	0	NA	NA	NA	NA
0	0	NA	NA	NA	NA
1	0	1	0,84894777	8	NA
0	0	1	-0,8086539	8	NA
0	0	NA	NA	NA	NA
0	0	NA	NA	NA	mod2_hub8
0	0	NA	NA	NA	NA
0	0	1	0,71408021	8	NA
0	0	NA	NA	NA	NA
0	0	1	0,892603	6	NA
0	0	NA	NA	NA	NA
0	0	NA	NA	NA	NA
0	0	NA	NA	NA	NA
0	0	NA	NA	NA	NA
0	0	NA	NA	NA	NA
0	0	NA	NA	NA	mod2_hub9
0	0	NA	NA	NA	NA
0	0	1	0,62314919	8	NA
0	0	NA	NA	NA	NA
0	0	NA	NA	NA	mod1_hub4
0	0	NA	NA	NA	NA
0	0	NA	NA	NA	NA
0	0	NA	NA	NA	NA
0	0	NA	NA	NA	NA
0	0	NA	NA	NA	NA
0	0	NA	NA	NA	NA
0	0	NA	NA	NA	NA
0	0	NA	NA	NA	NA
0	0	1	0,93888835	8	NA

0	0	NA	NA	NA	NA
0	0	NA	NA	NA	NA
0	0	NA	NA	NA	NA
0	0	NA	NA	NA	NA
0	0	NA	NA	NA	mod2_hub13
0	0	NA	NA	NA	NA
0	0	NA	NA	NA	NA
0	0	NA	NA	NA	NA
0	0	NA	NA	NA	NA
0	0	NA	NA	NA	NA
0	0	NA	NA	NA	NA
0	0	NA	NA	NA	NA
0	0	NA	NA	NA	NA
0	0	NA	NA	NA	NA
0	0	NA	NA	NA	NA
0	0	1	0,85190899	8	NA
0	0	NA	NA	NA	NA
0	0	NA	NA	NA	NA
0	0	NA	NA	NA	NA
0	0	NA	NA	NA	mod1_hub6
0	0	NA	NA	NA	NA
0	0	NA	NA	NA	NA
0	0	NA	NA	NA	NA
0	0	NA	NA	NA	NA
0	0	NA	NA	NA	NA
0	0	NA	NA	NA	NA
0	0	NA	NA	NA	NA
0	0	1	0,69777705	8	NA
0	0	NA	NA	NA	NA
0	0	NA	NA	NA	NA
0	0	NA	NA	NA	NA
0	0	NA	NA	NA	NA
0	0	NA	NA	NA	NA
0	0	NA	NA	NA	NA
0	0	1	0,78973256	8	NA
0	0	NA	NA	NA	NA
0	0	NA	NA	NA	NA
0	0	NA	NA	NA	NA
0	0	1	0,59597121	8	NA
0	0	NA	NA	NA	mod2_hub10
0	0	NA	NA	NA	NA
0	0	NA	NA	NA	NA
0	0	NA	NA	NA	mod2_hub14
0	0	NA	NA	NA	NA
0	0	NA	NA	NA	NA
0	0	NA	NA	NA	NA
0	0	NA	NA	NA	NA
1	1	NA	NA	NA	NA

1	1	NA	NA	NA	NA
1	1	NA	NA	NA	NA
1	1	NA	NA	NA	NA
1	1	NA	NA	NA	NA
1	1	NA	NA	NA	NA
0	0	NA	NA	NA	NA
0	0	NA	NA	NA	NA
0	0	1	-0,5951815	8	NA
0	0	1	1,03864802	8	NA
0	0	1	-0,6258896	8	NA
0	0	NA	NA	NA	NA
0	0	NA	NA	NA	NA
0	0	NA	NA	NA	NA
0	0	NA	NA	NA	NA
0	0	NA	NA	NA	NA
0	0	1	0,69436444	8	mod1_hub9
0	0	NA	NA	NA	NA
0	0	NA	NA	NA	NA
0	0	NA	NA	NA	NA
0	0	1	0,65139435	8	NA
0	0	NA	NA	NA	NA
0	0	NA	NA	NA	NA
0	0	1	1,4027006	8	NA
0	0	NA	NA	NA	NA
0	0	1	0,73063703	8	NA
0	0	NA	NA	NA	mod2_hub4
0	0	NA	NA	NA	NA
0	0	NA	NA	NA	NA
0	0	NA	NA	NA	NA
0	0	NA	NA	NA	NA
1	1	NA	NA	NA	NA
1	1	NA	NA	NA	NA
0	0	NA	NA	NA	NA
0	0	NA	NA	NA	NA
0	0	NA	NA	NA	NA
0	0	NA	NA	NA	NA
0	0	NA	NA	NA	mod2_hub2
0	0	NA	NA	NA	NA
0	0	1	0,71190829	6	NA
0	0	NA	NA	NA	NA
0	0	NA	NA	NA	mod1_hub8
1	1	NA	NA	NA	NA
0	0	NA	NA	NA	NA
0	0	1	1,32549545	8	mod1_hub7
0	0	NA	NA	NA	NA
0	0	NA	NA	NA	NA
0	0	NA	NA	NA	NA
0	0	NA	NA	NA	NA

0	0	NA	NA	NA	NA
1	0	NA	NA	NA	NA
0	0	1	1,22904468	8	NA
0	0	1	1,65013306	8	NA
0	0	NA	NA	NA	NA
0	0	1	1,36074948	8	NA
0	0	NA	NA	NA	NA
0	0	NA	NA	NA	NA
0	0	NA	NA	NA	NA
0	0	NA	NA	NA	NA
0	0	1	-0,6344073	8	NA
0	0	NA	NA	NA	NA
0	0	NA	NA	NA	mod1_hub2
0	0	NA	NA	NA	NA
0	0	NA	NA	NA	NA
0	1	NA	NA	NA	NA
0	1	NA	NA	NA	mod2_hub1
0	0	NA	NA	NA	NA
0	0	NA	NA	NA	NA
0	0	NA	NA	NA	NA
0	0	NA	NA	NA	NA
0	0	NA	NA	NA	NA
0	0	NA	NA	NA	mod1_hub5
0	0	NA	NA	NA	NA
0	0	NA	NA	NA	NA
0	0	NA	NA	NA	NA
0	0	NA	NA	NA	NA
