# Supplementary material for: Distinct transcriptional signatures in purified circulating immune cells drive heterogeneity in disease location in IBD
Source: BMJ Open Gastroenterol. 2023 Feb 6;10(1):e001003. doi: 10.1136/bmjgast-2022-001003 (PMC9906185; doi:10.1136/bmjgast-2022-001003)
Supplement: Supplementary data [file bmjgast-2022-001003supp007.pdf]

Rank	1-cos(α)	Perturbation	Compound_Summary	Cell-line
1	18.303	STK397047	http://pubchem.ncbi.nlm.nih.gov/su	A375
2	18.301	Chemistry	http://life.ccs.miami.edu/life/summ	SW620
3	18.293	BRD-A36630	http://pubchem.ncbi.nlm.nih.gov/su	SKLU1
4	18.261	BRD-K92317	http://pubchem.ncbi.nlm.nih.gov/su	PL21
5	18.196	PubChem CID	http://pubchem.ncbi.nlm.nih.gov/su	VCAP
6	18.157	withaferin-a	http://pubchem.ncbi.nlm.nih.gov/su	SKBR3
7	18.153	BRD-K04853	http://pubchem.ncbi.nlm.nih.gov/su	HT29
8	18.130	PubChem CID	http://pubchem.ncbi.nlm.nih.gov/su	VCAP
9	18.121	celastrol	http://pubchem.ncbi.nlm.nih.gov/su	HS578T
10	18.102	BRD-K04853	http://pubchem.ncbi.nlm.nih.gov/su	HT29
11	18.102	Narciclasine	http://pubchem.ncbi.nlm.nih.gov/su	HCC15
12	18.102	BRD-K92317	http://pubchem.ncbi.nlm.nih.gov/su	HCC515
13	18.093	BRD-K28916	http://pubchem.ncbi.nlm.nih.gov/su	A549
14	18.069	Ryuvidine	http://pubchem.ncbi.nlm.nih.gov/su	HCC515
15	18.066	withaferin-a	http://pubchem.ncbi.nlm.nih.gov/su	BT20
16	18.057	withaferin-a	http://pubchem.ncbi.nlm.nih.gov/su	SKBR3
17	18.055	F1566-0341	http://pubchem.ncbi.nlm.nih.gov/su	HT29
18	18.052	7241-4207	http://pubchem.ncbi.nlm.nih.gov/su	VCAP
19	18.039	STK249718	http://pubchem.ncbi.nlm.nih.gov/su	HCC515
20	18.030	BRD-K98824	http://pubchem.ncbi.nlm.nih.gov/su	A375
21	18.028	F1566-0341	http://pubchem.ncbi.nlm.nih.gov/su	VCAP
22	18.011	7241-4207	http://pubchem.ncbi.nlm.nih.gov/su	HT29
23	17.994	BRD-K77877	http://pubchem.ncbi.nlm.nih.gov/su	VCAP
24	17.994	BRD-K92317	http://pubchem.ncbi.nlm.nih.gov/su	A375
25	17.979	F1566-0341	http://pubchem.ncbi.nlm.nih.gov/su	MCF7
26	17.971	celastrol	http://pubchem.ncbi.nlm.nih.gov/su	BT20
27	17.960	BRD-K92317	http://pubchem.ncbi.nlm.nih.gov/su	RMUGS
28	17.950	B4313	http://pubchem.ncbi.nlm.nih.gov/su	VCAP
29	17.946	BRD-K80786	http://pubchem.ncbi.nlm.nih.gov/su	HT29
30	17.942	BRD-K28907	http://pubchem.ncbi.nlm.nih.gov/su	HCC15
31	17.934	Narciclasine	http://pubchem.ncbi.nlm.nih.gov/su	T3M10
32	17.933	BRD-K68143	http://pubchem.ncbi.nlm.nih.gov/su	HEPG2
33	17.928	TENIPOSIDE	http://pubchem.ncbi.nlm.nih.gov/su	SKLU1
34	17.924	YM-155	http://pubchem.ncbi.nlm.nih.gov/su	SW620
35	17.923	BRD-K91370	http://pubchem.ncbi.nlm.nih.gov/su	HT29
36	17.921	BRD-K99633	http://pubchem.ncbi.nlm.nih.gov/su	PC3
37	17.911	Narciclasine	http://pubchem.ncbi.nlm.nih.gov/su	SKLU1
38	17.904	QL-XII-47	http://pubchem.ncbi.nlm.nih.gov/su	HME1
39	17.888	VU0410183-	http://pubchem.ncbi.nlm.nih.gov/su	A375
40	17.885	celastrol	http://pubchem.ncbi.nlm.nih.gov/su	MDAMB231
41	17.876	withaferin-a	http://pubchem.ncbi.nlm.nih.gov/su	BT20
42	17.875	NCGC001885	http://pubchem.ncbi.nlm.nih.gov/su	VCAP
43	17.874	LDN-193189	http://pubchem.ncbi.nlm.nih.gov/su	HT29
44	17.867	BRD-K28916	http://pubchem.ncbi.nlm.nih.gov/su	A375
45	17.864	curcumin	http://pubchem.ncbi.nlm.nih.gov/su	HT29
46	17.864	BRD-K17140	http://pubchem.ncbi.nlm.nih.gov/su	A673
47	17.862	mocetinosta	http://pubchem.ncbi.nlm.nih.gov/su	A375

48	17.850	BRD-K99633(	http://pubchem.ncbi.nlm.nih.gov/su	HT29
49	17.839	mocetinostat	http://pubchem.ncbi.nlm.nih.gov/su	PC3
50	17.836	YM-155	http://pubchem.ncbi.nlm.nih.gov/su	A375

**Dose**	**Time**	**URL**
10.0um	6.0h	https://maayanlab.cloud/L1000CDS2/meta?sig_id=CPC014_A375_
10.0um	6.0h	https://maayanlab.cloud/L1000CDS2/meta?sig_id=CPC006_SW62
0.35um	6.0h	https://maayanlab.cloud/L1000CDS2/meta?sig_id=CPC006_SKLU1
10.0um	6.0h	https://maayanlab.cloud/L1000CDS2/meta?sig_id=CPC006_PL21_
10.0um	24.0h	https://maayanlab.cloud/L1000CDS2/meta?sig_id=CPC014_VCAP_
3.33um	24h	https://maayanlab.cloud/L1000CDS2/meta?sig_id=LJP005_SKBR3
10.0um	6.0h	https://maayanlab.cloud/L1000CDS2/meta?sig_id=CPC006_HT29_
10.0um	6.0h	https://maayanlab.cloud/L1000CDS2/meta?sig_id=CPC013_VCAP_
3.33um	24h	https://maayanlab.cloud/L1000CDS2/meta?sig_id=LJP006_HS578
10.0um	24.0h	https://maayanlab.cloud/L1000CDS2/meta?sig_id=CPC006_HT29_
10.0um	6.0h	https://maayanlab.cloud/L1000CDS2/meta?sig_id=CPC006_HCC15
10.0um	6.0h	https://maayanlab.cloud/L1000CDS2/meta?sig_id=CPC010_HCC51
10.0um	6.0h	https://maayanlab.cloud/L1000CDS2/meta?sig_id=CPC010_A549_
10.0um	6.0h	https://maayanlab.cloud/L1000CDS2/meta?sig_id=CPC001_HCC51
3.33um	24h	https://maayanlab.cloud/L1000CDS2/meta?sig_id=LJP005_BT20_
3.33um	24h	https://maayanlab.cloud/L1000CDS2/meta?sig_id=LJP006_SKBR3
10.0um	6.0h	https://maayanlab.cloud/L1000CDS2/meta?sig_id=CPC014_HT29_
10.0um	24.0h	https://maayanlab.cloud/L1000CDS2/meta?sig_id=CPC013_VCAP_
10.0um	6.0h	https://maayanlab.cloud/L1000CDS2/meta?sig_id=CPC014_HCC51
10.0um	6.0h	https://maayanlab.cloud/L1000CDS2/meta?sig_id=CPC019_A375_
10.0um	6.0h	https://maayanlab.cloud/L1000CDS2/meta?sig_id=CPC014_VCAP_
10.0um	6.0h	https://maayanlab.cloud/L1000CDS2/meta?sig_id=CPC013_HT29_
10.0um	24.0h	https://maayanlab.cloud/L1000CDS2/meta?sig_id=CPC020_VCAP_
10.0um	6.0h	https://maayanlab.cloud/L1000CDS2/meta?sig_id=CPC006_A375_
10.0um	6.0h	https://maayanlab.cloud/L1000CDS2/meta?sig_id=CPC014_MCF7
3.33um	24h	https://maayanlab.cloud/L1000CDS2/meta?sig_id=LJP006_BT20_
10.0um	6.0h	https://maayanlab.cloud/L1000CDS2/meta?sig_id=CPC006_RMUG
10.0um	24.0h	https://maayanlab.cloud/L1000CDS2/meta?sig_id=CPC014_VCAP_
10.0um	6.0h	https://maayanlab.cloud/L1000CDS2/meta?sig_id=CPC013_HT29_
11.1um	6.0h	https://maayanlab.cloud/L1000CDS2/meta?sig_id=CPC006_HCC15
10.0um	6.0h	https://maayanlab.cloud/L1000CDS2/meta?sig_id=CPC006_T3M1
10.0um	6.0h	https://maayanlab.cloud/L1000CDS2/meta?sig_id=CPC006_HEPG
1.25um	6.0h	https://maayanlab.cloud/L1000CDS2/meta?sig_id=CPC006_SKLU1
0.31um	6.0h	https://maayanlab.cloud/L1000CDS2/meta?sig_id=CPC006_SW62
10.0um	6.0h	https://maayanlab.cloud/L1000CDS2/meta?sig_id=CPC016_HT29_
10.0um	6.0h	https://maayanlab.cloud/L1000CDS2/meta?sig_id=CPC010_PC3_6
10.0um	6.0h	https://maayanlab.cloud/L1000CDS2/meta?sig_id=CPC006_SKLU1
3.33um	3h	https://maayanlab.cloud/L1000CDS2/meta?sig_id=LJP006_HME1_
10.0um	24.0h	https://maayanlab.cloud/L1000CDS2/meta?sig_id=CPC008_A375_
3.33um	24h	https://maayanlab.cloud/L1000CDS2/meta?sig_id=LJP006_MDAM
3.33um	24h	https://maayanlab.cloud/L1000CDS2/meta?sig_id=LJP006_BT20_
10.0um	6.0h	https://maayanlab.cloud/L1000CDS2/meta?sig_id=CPC007_VCAP_
10um	24h	https://maayanlab.cloud/L1000CDS2/meta?sig_id=LJP005_HT29_
10.0um	6.0h	https://maayanlab.cloud/L1000CDS2/meta?sig_id=CPC010_A375_
48.0um	24.0h	https://maayanlab.cloud/L1000CDS2/meta?sig_id=CPC006_HT29_
11.1um	6.0h	https://maayanlab.cloud/L1000CDS2/meta?sig_id=CPC006_A673_
10um	24h	https://maayanlab.cloud/L1000CDS2/meta?sig_id=LJP008_A375_

10.0um	6.0h	https://maayanlab.cloud/L1000CDS2/meta?sig_id=CPC010_HT29_
10um	24h	https://maayanlab.cloud/L1000CDS2/meta?sig_id=LJP008_PC3_2
0.31um	24.0h	https://maayanlab.cloud/L1000CDS2/meta?sig_id=CPC006_A375_

_6H:BRD-K80622725:10.0
0_6H:BRD-K74402642:10.0
._6H:BRD-A36630025:0.35
6H:BRD-K92317137:10.0
_24H:BRD-A52193669:10.0
_24H:BRD-K88378636:3.33
_6H:BRD-K04853698:10.0
_6H:BRD-K69852452:10.0
T_24H:BRD-A24396574:3.33
_24H:BRD-K04853698:10.0
_6H:BRD-K06792661:10.0
.5_6H:BRD-K92317137:10.0
_6H:BRD-K28916077:10.0
.5_6H:BRD-K06426971:10.0
24H:BRD-K88378636:3.33
_24H:BRD-K88378636:3.33
_6H:BRD-K69852452:10.0
_24H:BRD-K69852452:10.0
.5_6H:BRD-K81142122:10.0
_6H:BRD-K98824517:10.0
_6H:BRD-K69852452:10.0
_6H:BRD-K69852452:10.0
_24H:BRD-K77877933:10.0
_6H:BRD-K92317137:10.0
_6H:BRD-K69852452:10.0
24H:BRD-A24396574:3.33
GS_6H:BRD-K92317137:10.0
_24H:BRD-A11007541:10.0
_6H:BRD-K80786583:10.0
_6H:BRD-K28907958:11.1
0_6H:BRD-K06792661:10.0
2_6H:BRD-K68143200:10.0
._6H:BRD-A35588707:1.25
0_6H:BRD-K76703230:0.31
_6H:BRD-K91370081:10.0
H:BRD-K99633092:10.0
._6H:BRD-K06792661:10.0
_3H:BRD-K99252563:3.33
_24H:BRD-K74710236:10.0
IB231_24H:BRD-A24396574:3.33
24H:BRD-K88378636:3.33
_6H:BRD-A09719808:10.0
24H:BRD-K04853698:10
_6H:BRD-K28916077:10.0
_24H:BRD-K74148702:48.0
_6H:BRD-K17140735:11.1
24H:BRD-K16485616:10

_6H:BRD-K99633092:10.0
4H:BRD-K16485616:10
_24H:BRD-K76703230:0.31
