# Supplementary material for: Distinct transcriptional signatures in purified circulating immune cells drive heterogeneity in disease location in IBD
Source: BMJ Open Gastroenterol. 2023 Feb 6;10(1):e001003. doi: 10.1136/bmjgast-2022-001003 (PMC9906185; doi:10.1136/bmjgast-2022-001003)
Supplement: Supplementary data [file bmjgast-2022-001003supp008.pdf]

Rank	1-cos(α)	Perturbation
1	0.2305	BMS-387032
2	0.2490	A443654
3	0.2540	JNK-9L
4	0.2558	alvocidib
5	0.2559	A443654
6	0.2561	PF-562271
7	0.2578	AT-7519
8	0.2630	alvocidib
9	0.2684	linifanib
10	0.2706	alvocidib
11	0.2777	A443654
12	0.2786	alvocidib
13	0.2803	CGP-60474
14	0.2808	A443654
15	0.2832	pelitinib
16	0.2838	CGP-60474
17	0.2846	CGP-60474
18	0.2860	AS-601245
19	0.2862	CGP-60474
20	0.2865	BMS-387032
21	0.2894	alvocidib
22	0.2933	CGP-60474
23	0.2949	alvocidib
24	0.2960	alvocidib
25	0.2972	PHA-793887
26	0.2980	16-HYDROXY
27	0.2985	AZD-5438
28	0.3001	daunorubicin
29	0.3050	alvocidib
30	0.3075	AT-7519
31	0.3084	BMS-387032
32	0.3087	alvocidib
33	0.3088	AT-7519
34	0.3097	alvocidib
35	0.3102	BMS-387032
36	0.3139	CGP-60474
37	0.3148	alvocidib
38	0.3152	JNK-9L
39	0.3156	CGP-60474
40	0.3168	JNK-9L
41	0.3183	JNK-9L
42	0.3191	CGP-60474
43	0.3205	CGP-60474
44	0.3208	A443654
45	0.3220	AT-7519
46	0.3223	CGP-60474
47	0.3232	AT-7519

48	0.3239	A443654
49	0.3245	CGP-60474
50	0.3251	BMS-387032

Compound_Summary	Cell-line
http://pubchem.ncbi.nlm.nih.gov/summary/summary.cgi?cid=3025986	HME1
None	HME1
http://pubchem.ncbi.nlm.nih.gov/summary/summary.cgi?cid=59588070	MDAMB231
http://pubchem.ncbi.nlm.nih.gov/summary/summary.cgi?cid=5287969	HME1
None	MCF10A
http://pubchem.ncbi.nlm.nih.gov/summary/summary.cgi?cid=11713159	HME1
http://pubchem.ncbi.nlm.nih.gov/summary/summary.cgi?cid=11338033	MDAMB231
http://pubchem.ncbi.nlm.nih.gov/summary/summary.cgi?cid=5287969	MDAMB231
http://pubchem.ncbi.nlm.nih.gov/summary/summary.cgi?cid=11485656	HME1
http://pubchem.ncbi.nlm.nih.gov/summary/summary.cgi?cid=5287969	BT20
None	HS578T
http://pubchem.ncbi.nlm.nih.gov/summary/summary.cgi?cid=5287969	HME1
http://pubchem.ncbi.nlm.nih.gov/summary/summary.cgi?cid=644215	HME1
None	MDAMB231
http://pubchem.ncbi.nlm.nih.gov/summary/summary.cgi?cid=6445562	MDAMB231
http://pubchem.ncbi.nlm.nih.gov/summary/summary.cgi?cid=644215	HME1
http://pubchem.ncbi.nlm.nih.gov/summary/summary.cgi?cid=644215	HME1
http://pubchem.ncbi.nlm.nih.gov/summary/summary.cgi?cid=10109823	HS578T
http://pubchem.ncbi.nlm.nih.gov/summary/summary.cgi?cid=644215	HME1
http://pubchem.ncbi.nlm.nih.gov/summary/summary.cgi?cid=3025986	HME1
http://pubchem.ncbi.nlm.nih.gov/summary/summary.cgi?cid=5287969	HME1
http://pubchem.ncbi.nlm.nih.gov/summary/summary.cgi?cid=644215	MDAMB231
http://pubchem.ncbi.nlm.nih.gov/summary/summary.cgi?cid=5287969	BT20
http://pubchem.ncbi.nlm.nih.gov/summary/summary.cgi?cid=5287969	HME1
http://pubchem.ncbi.nlm.nih.gov/summary/summary.cgi?cid=46191454	HME1
http://pubchem.ncbi.nlm.nih.gov/summary/summary.cgi?cid=16220015	SKMEL1
http://pubchem.ncbi.nlm.nih.gov/summary/summary.cgi?cid=16747683	BT20
http://pubchem.ncbi.nlm.nih.gov/summary/summary.cgi?cid=3085107	PC3
http://pubchem.ncbi.nlm.nih.gov/summary/summary.cgi?cid=5287969	BT20
http://pubchem.ncbi.nlm.nih.gov/summary/summary.cgi?cid=11338033	HME1
http://pubchem.ncbi.nlm.nih.gov/summary/summary.cgi?cid=3025986	BT20
http://pubchem.ncbi.nlm.nih.gov/summary/summary.cgi?cid=5287969	HS578T
http://pubchem.ncbi.nlm.nih.gov/summary/summary.cgi?cid=11338033	MDAMB231
http://pubchem.ncbi.nlm.nih.gov/summary/summary.cgi?cid=5287969	HME1
http://pubchem.ncbi.nlm.nih.gov/summary/summary.cgi?cid=3025986	HS578T
http://pubchem.ncbi.nlm.nih.gov/summary/summary.cgi?cid=644215	HS578T
http://pubchem.ncbi.nlm.nih.gov/summary/summary.cgi?cid=5287969	HS578T
http://pubchem.ncbi.nlm.nih.gov/summary/summary.cgi?cid=59588070	HME1
http://pubchem.ncbi.nlm.nih.gov/summary/summary.cgi?cid=644215	HS578T
http://pubchem.ncbi.nlm.nih.gov/summary/summary.cgi?cid=59588070	BT20
http://pubchem.ncbi.nlm.nih.gov/summary/summary.cgi?cid=59588070	LNCAP
http://pubchem.ncbi.nlm.nih.gov/summary/summary.cgi?cid=644215	MDAMB231
http://pubchem.ncbi.nlm.nih.gov/summary/summary.cgi?cid=644215	MDAMB231
None	LNCAP
http://pubchem.ncbi.nlm.nih.gov/summary/summary.cgi?cid=11338033	HME1
http://pubchem.ncbi.nlm.nih.gov/summary/summary.cgi?cid=644215	HS578T
http://pubchem.ncbi.nlm.nih.gov/summary/summary.cgi?cid=11338033	HME1

None	HME1
http://pubchem.ncbi.nlm.nih.gov/summary/summary.cgi?cid=644215	BT20
http://pubchem.ncbi.nlm.nih.gov/summary/summary.cgi?cid=3025986	HS578T

Dose	Time	URL
0.12um	3h	https://maayanlab.cloud/L1000CDS2/meta?sig_id=LJP006_HME1_
1.11um	3h	https://maayanlab.cloud/L1000CDS2/meta?sig_id=LJP006_HME1_
3.33um	3h	https://maayanlab.cloud/L1000CDS2/meta?sig_id=LJP006_MDAM
0.04um	3h	https://maayanlab.cloud/L1000CDS2/meta?sig_id=LJP006_HME1_
3.33um	3h	https://maayanlab.cloud/L1000CDS2/meta?sig_id=LJP006_MCF10
10um	3h	https://maayanlab.cloud/L1000CDS2/meta?sig_id=LJP006_HME1_
0.37um	3h	https://maayanlab.cloud/L1000CDS2/meta?sig_id=LJP006_MDAM
0.04um	3h	https://maayanlab.cloud/L1000CDS2/meta?sig_id=LJP006_MDAM
10um	3h	https://maayanlab.cloud/L1000CDS2/meta?sig_id=LJP006_HME1_
0.37um	3h	https://maayanlab.cloud/L1000CDS2/meta?sig_id=LJP006_BT20_
3.33um	3h	https://maayanlab.cloud/L1000CDS2/meta?sig_id=LJP006_HS578
3.33um	3h	https://maayanlab.cloud/L1000CDS2/meta?sig_id=LJP006_HME1_
1.11um	3h	https://maayanlab.cloud/L1000CDS2/meta?sig_id=LJP006_HME1_
3.33um	3h	https://maayanlab.cloud/L1000CDS2/meta?sig_id=LJP006_MDAM
3.33um	3h	https://maayanlab.cloud/L1000CDS2/meta?sig_id=LJP005_MDAM
0.04um	3h	https://maayanlab.cloud/L1000CDS2/meta?sig_id=LJP006_HME1_
0.12um	3h	https://maayanlab.cloud/L1000CDS2/meta?sig_id=LJP006_HME1_
3.33um	3h	https://maayanlab.cloud/L1000CDS2/meta?sig_id=LJP005_HS578
0.37um	3h	https://maayanlab.cloud/L1000CDS2/meta?sig_id=LJP006_HME1_
1.11um	3h	https://maayanlab.cloud/L1000CDS2/meta?sig_id=LJP006_HME1_
1.11um	3h	https://maayanlab.cloud/L1000CDS2/meta?sig_id=LJP006_HME1_
3.33um	3h	https://maayanlab.cloud/L1000CDS2/meta?sig_id=LJP006_MDAM
0.12um	3h	https://maayanlab.cloud/L1000CDS2/meta?sig_id=LJP006_BT20_
0.12um	3h	https://maayanlab.cloud/L1000CDS2/meta?sig_id=LJP006_HME1_
10um	3h	https://maayanlab.cloud/L1000CDS2/meta?sig_id=LJP006_HME1_
0.08um	6.0h	https://maayanlab.cloud/L1000CDS2/meta?sig_id=CPC006_SKME
3.33um	3h	https://maayanlab.cloud/L1000CDS2/meta?sig_id=LJP006_BT20_
10.0um	6.0h	https://maayanlab.cloud/L1000CDS2/meta?sig_id=CPC009_PC3_6
3.33um	3h	https://maayanlab.cloud/L1000CDS2/meta?sig_id=LJP006_BT20_
3.33um	3h	https://maayanlab.cloud/L1000CDS2/meta?sig_id=LJP006_HME1_
3.33um	3h	https://maayanlab.cloud/L1000CDS2/meta?sig_id=LJP006_BT20_
3.33um	3h	https://maayanlab.cloud/L1000CDS2/meta?sig_id=LJP006_HS578
1.11um	3h	https://maayanlab.cloud/L1000CDS2/meta?sig_id=LJP006_MDAM
10um	3h	https://maayanlab.cloud/L1000CDS2/meta?sig_id=LJP006_HME1_
1.11um	3h	https://maayanlab.cloud/L1000CDS2/meta?sig_id=LJP006_HS578
0.37um	3h	https://maayanlab.cloud/L1000CDS2/meta?sig_id=LJP005_HS578
0.12um	3h	https://maayanlab.cloud/L1000CDS2/meta?sig_id=LJP006_HS578
10um	3h	https://maayanlab.cloud/L1000CDS2/meta?sig_id=LJP006_HME1_
0.12um	3h	https://maayanlab.cloud/L1000CDS2/meta?sig_id=LJP006_HS578
10um	3h	https://maayanlab.cloud/L1000CDS2/meta?sig_id=LJP006_BT20_
3.33um	3h	https://maayanlab.cloud/L1000CDS2/meta?sig_id=LJP006_LNCAP
0.12um	3h	https://maayanlab.cloud/L1000CDS2/meta?sig_id=LJP006_MDAM
1.11um	3h	https://maayanlab.cloud/L1000CDS2/meta?sig_id=LJP006_MDAM
1.11um	3h	https://maayanlab.cloud/L1000CDS2/meta?sig_id=LJP006_LNCAP
0.37um	3h	https://maayanlab.cloud/L1000CDS2/meta?sig_id=LJP006_HME1_
1.11um	3h	https://maayanlab.cloud/L1000CDS2/meta?sig_id=LJP006_HS578
1.11um	3h	https://maayanlab.cloud/L1000CDS2/meta?sig_id=LJP006_HME1_

3.33um	3h	https://maayanlab.cloud/L1000CDS2/meta?sig_id=LJP006_HME1_
0.37um	3h	https://maayanlab.cloud/L1000CDS2/meta?sig_id=LJP006_BT20_
0.12um	3h	https://maayanlab.cloud/L1000CDS2/meta?sig_id=LJP006_HS578

_3H:BRD-K43389698:0.12
_3H:BRD-K88573743:1.11
IB231_3H:BRD-K19220233:3.33
_3H:BRD-K87909389:0.04
)A_3H:BRD-K88573743:3.33
_3H:BRD-K99545815:10
IB231_3H:BRD-K13390322:0.37
IB231_3H:BRD-K87909389:0.04
_3H:BRD-K99749624:10
3H:BRD-K87909389:0.37
T_3H:BRD-K88573743:3.33
_3H:BRD-K87909389:3.33
_3H:BRD-K79090631:1.11
IB231_3H:BRD-K88573743:3.33
IB231_3H:BRD-K08799216:3.33
_3H:BRD-K79090631:0.04
_3H:BRD-K79090631:0.12
T_3H:BRD-A60245366:3.33
_3H:BRD-K79090631:0.37
_3H:BRD-K43389698:1.11
_3H:BRD-K87909389:1.11
IB231_3H:BRD-K79090631:3.33
3H:BRD-K87909389:0.12
_3H:BRD-K87909389:0.12
_3H:BRD-K64800655:10
L1_6H:BRD-A13122391:0.08
3H:BRD-K72414522:3.33
iH:BRD-A68009927:10.0
3H:BRD-K87909389:3.33
_3H:BRD-K13390322:3.33
3H:BRD-K43389698:3.33
T_3H:BRD-K87909389:3.33
IB231_3H:BRD-K13390322:1.11
_3H:BRD-K87909389:10
T_3H:BRD-K43389698:1.11
T_3H:BRD-K79090631:0.37
T_3H:BRD-K87909389:0.12
_3H:BRD-K19220233:10
T_3H:BRD-K79090631:0.12
3H:BRD-K19220233:10
_3H:BRD-K19220233:3.33
IB231_3H:BRD-K79090631:0.12
IB231_3H:BRD-K79090631:1.11
_3H:BRD-K88573743:1.11
_3H:BRD-K13390322:0.37
T_3H:BRD-K79090631:1.11
_3H:BRD-K13390322:1.11

_3H:BRD-K88573743:3.33
3H:BRD-K79090631:0.37
T_3H:BRD-K43389698:0.12
